# Procollagen-lysine 2-oxoglutarate 5-dioxygenases are responsible for 5R-hydroxylysine modification of therapeutic T-cell bispecific monoclonal antibodies produced by Chinese hamster ovary cells

**DOI:** 10.3389/fbioe.2024.1414408

**Published:** 2024-10-28

**Authors:** Niels Bauer, Marco Boettger, Styliani Papadaki, Tanja Leitner, Stefan Klostermann, Hubert Kettenberger, Guy Georges, Vincent Larraillet, Dino Gluhacevic von Kruechten, Lars Hillringhaus, Annette Vogt, Simon Ausländer, Oliver Popp

**Affiliations:** ^1^ Large Molecule Research, Roche Pharma Research and Early Development (pRED), Roche Innovation Center Munich, Penzberg, Germany; ^2^ Gene Center and Department of Biochemistry, Ludwig-Maximilians-Universität München, Munich, Germany; ^3^ Data and Analytics, Roche Pharma Research and Early Development (pRED), Roche Innovation Center Munich, Penzberg, Germany; ^4^ Special Chemistry, Roche Diagnostics, Roche Innovation Center Munich, Penzberg, Germany

**Keywords:** T-cell bispecific monoclonal antibodies, Chinese hamster ovary cells, hydroxylysine, CRISPR/Cas9, post-translational modification, mass spectrometry, metal cofactor

## Abstract

We present a detailed mass spectrometric analysis of three 2 + 1 T-cell bispecific monoclonal antibodies (TCB mAbs), where an unexpected +15.9950 Da mass shift in tryptic peptides was observed. This modification was attributed to the occurrence of 5R-hydroxylysine (Hyl) using a hybrid LC–MS/MS molecular characterization and CRISPR/Cas9 gene deletion approach. The modification was found at various sites within TCB mAbs, with a conspicuous hot spot motif mirroring a prior observation where Hyl was mapped to the C_H_1–VH Fab domain interface of IgGs. In contrast to the preceding report, our structural modeling analysis on TCB mAbs unveiled substantial differences in the orientation and flexibility of motifs in immediate proximity and across the artificial C_H_1–VL cross Fab interface and upstream elbow segment. Utilizing a hybrid database search, RNAseq, and a CRISPR/Cas9 knockout methodology in Chinese hamster ovary (CHO) production cell lines, procollagen-lysine, 2-oxoglutarate 5-dioxygenases (PLODs) were conclusively identified as the catalyzing enzymes accountable for the 5R-Hyl modification in TCB mAbs. To quantitatively inhibit Hyl formation in TCB mAbs, the activity of all three Chinese hamster PLOD isoenzymes needs to be depleted via CRISPR/Cas9 gene knockout. Moreover, our investigation identified cell culture iron availability, process duration, and clonal variability in CHO cells as elements influencing the levels of Hyl formation in TCB mAbs. This research offers a solution for circumventing Hyl formation in therapeutic complex mAb formats, such as TCB mAbs, produced in CHO cell culture processes, thereby addressing potential technical and biological challenges associated with unintended Hyl modification.

## Introduction

**FIGURE 1:**
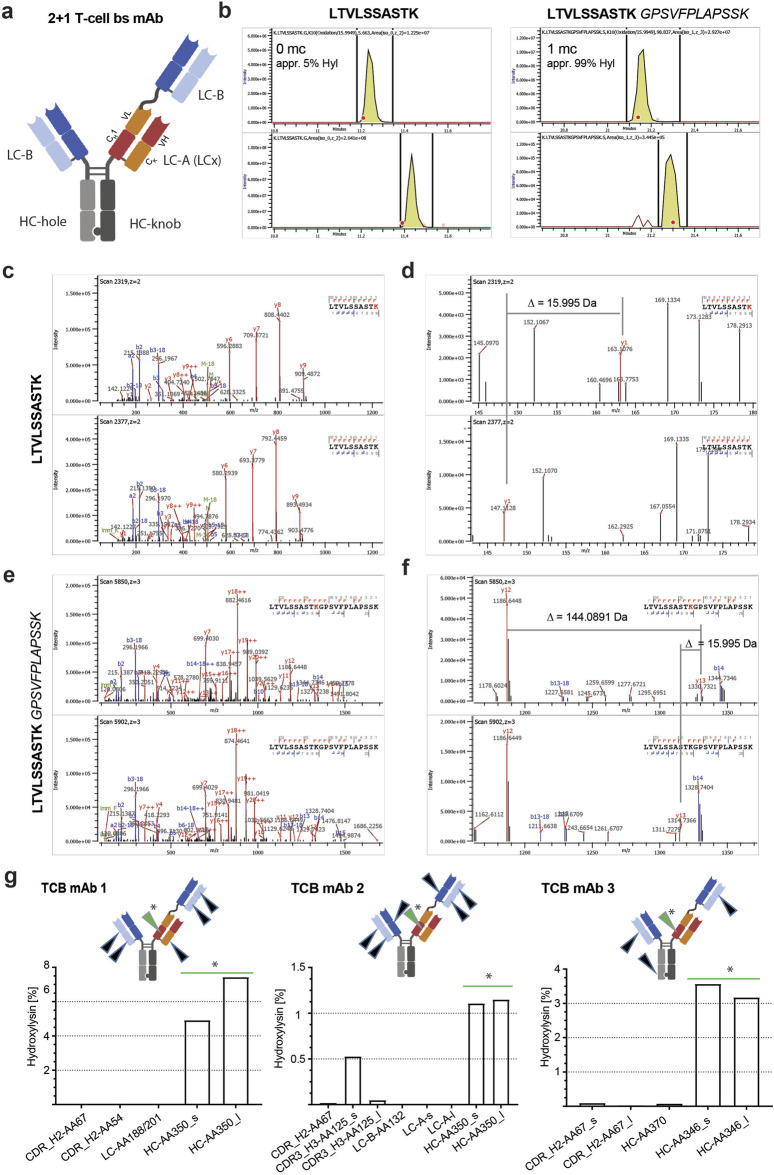
Conformation of hydroxylysine modification and relevance for tryptic digestion. **(A)** Schematic model of a 2 + 1 T-cell bispecific (TCB) mAb (dotted lines: disulfide bridge). **(B)** Example XIC of modified (upper panel) and unmodified (bottom panel) tryptic peptide HC-AA350 of TCB mAb 3 with zero missed cleavage LTVLSSASTK and the tryptic peptide with one missed cleavage LTVLSSASTKGPSVFLAPSSK. The bold red dots indicate the timepoints when the MS/MS fragmentations of the precursors have been triggered, which represent the main species of the respective XIC signals. Note the difference in the intensity scale of both unmodified peptides compared to the respective modified peptides. **(C)** Overview of HCD fragment spectra of the modified (upper panel) and unmodified peptide (bottom panel) HC-AA350 of TCB mAb 3. **(D)** Zoomed view of modified (upper panel) and unmodified peptides (lower panel). The shift of 15.9950 Da of the y1-ion confirms the modification on lysine. **(E)** Example overview of HCD fragment spectra of the modified (upper panel) and unmodified peptides (bottom panel) with one missed cleavage. **(F)** Zoomed view of the same MS/MS scans with a focus on the fragment ions y12 and y13 that belong to the KG motif. The distance between both fragments is equivalent to the mass of a Hyl residue with −5.6 ppm deviation (theoretical mass of Hyl = 144.0899). **(G)** Identified sites and levels of cleaved and missed cleaved tryptic peptides of three different TCB mAb molecules (arrowheads indicate the Hyl modification site in respective TCB molecule: green arrowhead and star represent hot spot positions and black arrowhead represents alternative positions).

Most recombinant generic monoclonal antibodies (mAbs) and novel, complex mAb-derived formats approved or under clinical investigation for the treatment of diverse therapeutic purposes are produced by mammalian Chinese hamster ovary (CHO) cells (Wurm, 2004). Innovative, complex mAb derivatives, such as T-cell bispecific (TCB) mAbs, exhibit potential therapeutic efficacies by orchestrating T-cell cytotoxicity toward pathogenic cells (Figure 1A) (Augsberger et al., 2021; Iurlaro et al., 2022). CHO cells, as the most prominent representative of mammalian expression systems, are preferred over other hosts due to their ability to grow in suspension at large scales in serum-free and chemically defined media and their capacity to produce high quantities of recombinant biotherapeutic proteins required to meet clinical demands. Importantly, CHO cells have demonstrated the ability to produce recombinant proteins with correct protein folding and human-tolerant post-translational modifications (PTMs), which are essential for clinical applications (Kunert and Reinhart, 2016). Significant investments in developing robust production strategies and metabolically balanced media formulations have enabled processes with high yields and product quality, as well as substantially improved batch-to-batch reproducibility (Birch and Racher, 2006). Despite the implementation of rigorous control strategies in the production of biologics, these proteins still exhibit minor variations that arise from both enzymatic functions and non-enzymatic chemical reactions during the production process. These micro heterogeneities include N- and O-types of glycosylation, cysteine modifications, carbonylations, oxidations, glycation, isomerizations of aspartate, and variations at the C-terminal lysine (Geist et al., 2013; Luo et al., 2012; Raju and Jordan, 2012; Gramer, 2014). In recent studies, an unanticipated modification of lysine hydroxylation was detected in various recombinant proteins produced by CHO cells, including tissue plasminogen activator (rtPA), soluble and chimeric CD4 receptor variants, the De13a toxin from a marine cone snail, somatostatin, and IgG1 monoclonal antibodies, all of which are present in significant quantities (Table 1) (Molony et al., 1995; Aguilar et al., 2005; Andrews et al., 1984; Xie et al., 2016). The hydroxylation in the recombinant IgG1 mAb has been identified by a +16 Da mass shift and is comparable to the other hydroxylated proteins in a Xaa-Lys-Gly (XKG) consensus sequence via a tryptic fragmentation and liquid chromatography–mass spectrometry approach (Xie et al., 2016).

**TABLE 1:** Hyl-containing therapeutic peptides and proteins reported.

Peptide/protein	Sequence	Level of Hyl	Reference
Recombinant human plasminogen activator (rtPA)	I**K**GGLFADIASHPWQAAIFAK	Up to 20%	Molony et al. (1995)
Recombinant antibody CD4/rCD4IgG	ILGNEGSFLT**K**GFSK	Up to 10%	Molony et al. (1995)
De13a, a toxin of marine cone snail	DCPTSCPTTCANGWECC**K**GYPCVN**K**ACSGCTH	Up to 100%	Aguilar et al. (2005)
Somatostatin-28, a peptide hormone from anglerfish	RSVDSTNNLPPRERKAGCKNFYW**K**GFTSC	Up to 40%	Andrews et al. (1984)
IgG1 mAb	XXXXXXXXXWGQGTLVTVSSAST**K**	Not assessed	Xie et al. (2016)
TCB mAb 1	XXXXXXXXXXXX**K**GR XXXXXXXXXXXX**K** DSTYSLSSTLTLS**K** LTVLSSAST**K**GPSVFPLAPSSK	12.4%	This work
TCB mAb 2	XXXXXXXXXXXX**K**GR XXXXXXWGQGVLVTVSSAST**K**GPSVFPLAPSSK L**K**SGTASVVCLLNNFYPR DSTYSLSSTLTLS**K**ADYEK LTVLSSAST**K**GPSVFPLAPSSK	2.9%	This work
TCB mAb 3	XXXXXXXXXXX**K**GR NQVSLSCAV**K** LTVLSSAST**K**GPSVFPLAPSSK	7.0%	This work

The red lysine represents the position of Hyl modification. Please note that “X” represents amino acids of the identified peptides from the mAb CDR regions.

In nature, enzymatic hydroxylysine (Hyl) formation by lysyl hydroxylases is a vital upstream key element in extracellular matrix reconstitution by crosslinking pro-collagen and collagen-like structures by O-glycosylation (Pornprasertsuk et al., 2004; Schegg et al., 2009). Pro-collagen protein Hyl formation in humans is facilitated by procollagen-lysine, 2-oxoglutarate 5-dioxygenases (PLOD) in the endoplasmatic reticulum (ER), which belong to the enzymatic class of 2-oxoglutarate (2OG) oxygenases (Markolovic et al., 2015). In humans, three genes, namely, *PLOD1*, *PLOD2*, and *PLOD3*, encode for protein lysyl hydroxylases, which catalyze the 5R-Hyl formation in pro-collagen and collagen-like proteins. For PLOD2, two splice variants, namely, LH2a (PLOD2A) and LH2b (PLOD2B) exist, where LH2b differs from LH2a by incorporating the small exon 13A (Valtavaara et al., 1997). The variety of PLOD gene products and splice variants suggest different layers of regulation in (patho)physiological processes; however, the role of the diverse gene products and splice variants is not fully understood yet (Qi and Xu, 2018). In mammalian systems, for example, a multiprotein complex containing the lysyl hydroxylase *PLOD1* and the proline hydroxylases P3H3 and P3H4 is responsible for the hydroxylation of C5 of lysyl and the C4 prolyl residues in pro-collagen α-chains, respectively, which subsequently triggers the accurate assembly and crosslinking of collagen fibrils (Heard et al., 2016).

Facilitating the enzymatic oxygenase activity, PLOD enzymes require Fe^2+^ as a cofactor and 2OG. In a sequential binding mechanism of the first 2OG, the substrate, and then oxygen, an active ferryl intermediate is formed by oxidative decarboxylation of 2OG. The ferryl intermediate finally reacts with the substrate, leading to hydroxylation. The proteinogenic substrates represent a generic XKG consensus sequence that targets the activity of oxygenases to specific protein surface features. PLODs differ from the other mammalian lysyl 2OGs of the Jumonji domain-containing (JmjC) protein family, like JMJD4, JMJD6, and JMJD7, due to differences in their subcellular spatial localization, respective downstream control mechanisms, and in generating isobar yet structurally different Hyl products: 5R-Hyl (PLODs), 5S-Hyl (JMJD6), 4RS-Hyl (JMJD4), and 3S-Hyl (JMJD7) (Markolovic et al., 2015; Markolovic et al., 2018).

The presence of the Hyl moiety in proteins alters their physicochemical properties since the additional hydroxyl group can cause local changes in hydrophobicity and charge and can act as an accessible active group for subsequent modifications like glycosylation [reviewed by De Giorgi et al. (2021)]. Such alterations have the potential to act as intended or unintended signaling modulators in biological systems and may induce undesirable complexity in the technical development and production of therapeutic proteins. Since Hyl residues are utilized for crosslinking in collagens, the accidental aggregation and/or neoepitope formation of Hyl in modified therapeutic proteins poses a risk to protein stability and functionality. For example, this may modulate target protein binding efficacy, increase the efforts required for analytical characterization and purification process development, and, ultimately, trigger immunogenicity in patients. In addition, Hyl can be targeted by lysyl oxidases to form hydroxyallysine through oxidative deamination, releasing ammonia and the strong oxidizing reagent H_2_O_2_ or serve as a motive for downstream O-glycosylation events (Abbasov et al., 2021). Therefore, once identified, detailed molecular and functional characterization of this PTM in biotherapeutics is required, as well as ways to control it. In an optimal situation, measures should be developed to avoid the *de novo* formation of Hyl modifications.

In this work, we report the identification of 5R-Hyl as a PTM in three different TCB mAbs produced by CHO cell culture processes via LC–MS/MS peptide mapping. In recent studies, CHO endogenous lysyl hydroxylase enzymes were postulated to mediate the formation of Hyl in mAbs. Using CRISPR/Cas9 knockout approaches, we demonstrate for the first time that all existing host-derived *Cricetulus griseus* PLOD isozymes, namely, PLOD1, PLOD2, and PLOD3, are responsible for the formation of the 5-Hyl modification of three recombinant TCB mAbs in CHO-based expression systems. Unexpectedly, the deletion of the PLOD genes in the respective recombinant CHO production cell lines correlated with significant benefits in cell culture performance and an increase in the product quality of the TCB mAbs in the harvested cell culture fluids.

## Results

### Identification of hydroxylysine modification in TCB mAbs

Using a spectrometric tryptic peptide mapping approach, we identified several +15.9950 Da mass shifts in three different recombinant TCB mAbs, namely, TCB mAb 1, TCB mAb 2, and TCB mAb 3, each expressed by three different recombinant CHO cell lines (Figures 1A, B). The levels of the peptide modifications varied based on the positions in the TCB mAb molecule (Table 2). The mass shifts were observed in the respective CDRs at low levels (<1%) and high levels in the C_H_1 heavy-chain knob (HC-K) backbone (up to 12.4%) downstream of the intramolecular crossed Fab CDR (Table 2; Figure 1G). The detected motif with the +15.9950 Da modification in the prominent C_H_1 mAb element is similar to the position described earlier by Xie et al. (2016) at a lysine residue in HC101–HC124 (XXXXXXXXXWGQGTLVTVSSASTK), yet differs in the present study by the crossed HC organization in TCB mAbs by introducing an artificial C_H_1–VL interface (Figure 1A). Xie et al. (2016) attributed the +15.9950 Da modification to the hydroxylation of the lysine residue in the identified peptide. Based on this previous observation, we aimed to identify the cause of observed mass shifts and evaluate the possibility of the Hyl modification in the three analyzed TCB mAbs. For this, the exact amino acid positions of the modifications were determined by the analysis of the higher-energy collisional dissociation (HCD) fragmentation data (Table 2). The location of the +15.9950 Da modification in the prominent LTVLSSASTK peptide (amino acids 341–350 according to HC position numbering–amino acid 350 corresponds to 117 according to Kabat numbering) of HC-K was attributed to oxidation and confirmed at lysine K350 in the analyzed peptide using extracted ion chromatograms of the modified and the unmodified peptide LTVLSSASTK350 for both cases with and without missed cleavage (Figure 1C). The hydroxylation of lysine results in a decreased hydrophobicity of the tryptic peptide with an expected earlier retention time compared to the unmodified peptide, which is confirmed by the XIC comparison of both and is in line with data, as previously reported by Xie et al. (2016) (Figure 1B).

**TABLE 2:** Hyl levels of TCBs produced by wild-type (wt) and PLOD KO production cell lines.

TCB mAb 1											
TCB mAb domain	Cleaved	Sequence	Protein annotation	Start AA	End AA	Mod. AAs	Mod. names	Var. Pos. Protein	TCB mAb 1 wt [%]	TCB mAb 1 KO [%]	Hyl reduction [%]
LC-B	nd	XXXXXXXXXXXX**K**GR	CDR-H2	55	69	K	Oxidation/15.9949	67	<0.1	<0.1	79^*^
LC-B	nd	XXXXXXXXXXXX**K**	CDR-H2	53	67	K	Oxidation/15.9949	54	<0.1	<0.1	84^*^
LC-A & B	nd	DSTYSLSSTLTLS**K**		175/188	188/201	K	Oxidation/15.9949	188/201	<0.1	<0.1	91^*^
HC-K	s	LTVLSSAST**K**		341	350	K	Oxidation/15.9949	350	4.9	0.2	93
HC-K	l	LTVLSSAST**K**GPSVFPLAPSSK		341	362	K	Oxidation/15.9949	350	7.4	0.7	
								Sum	12.3	0.9	

TCB mAb 2											
TCB mAb domain		Sequence	Protein annotation	Start AA	End AA	Mod. AAs	Mod. names	Var. Pos. Protein	TCB mAb 2 wt [%]	TCB mAb 2 KO [%]	Hyl reduction [%]
LC-A	nd	XXXXXXXXXXXX**K**GR	CDR-H2	55	69	K	Oxidation/15.9949	67	<0.1	<0.1	65^*^
HC-K	s	XXXXXXWGQGVLVTVSSAST**K**	CDR-H3	104	125	K	Oxidation/15.9949	125	0.5	<0.1	92
HC-K	l	XXXXXXWGQGVLVTVSSAST**K** GPSVFPLAPSSK	CDR-H3	104	137	K	Oxidation/15.9949	125	<0.1	<0.1	
								Sum	0.6	<0.1	
LC-B	nd	L**K**SGTASVVCLLNNFYPR		131	148	K	Oxidation/15.9949	132	<0.1	<0.1	96^*^
LC-A & B	s	DSTYSLSSTLTLS**K**		188/176	201/189	K	Oxidation/15.9949	201/189	<0.1	<0.1	98^*^
LC-A & B	l	DSTYSLSSTLTLS**K**ADYEK		188/176	206/198	K	Oxidation/15.9949	201/198	<0.1	<0.1	
								Sum	<0.1	<0.1	
HC-K	s	LTVLSSAST**K**		341	350	K	Oxidation/15.9949	350	1.1	<0.1	98
HC-K	l	LTVLSSAST**K**GPSVFPLAPSSK		341	362	K	Oxidation/15.9949	350	1.2	<0.1	
								Sum	2.3	<0.1	

TCB mAb 3											
TCB mAb domain		Sequence	Protein annotation	Start AA	End AA	Mod. AAs	Mod. names	Var. Pos. Protein	TCB mAb 3 wt [%]	TCB mAb 3 KO [%]	Hyl reduction [%]
LC-A	s	XXXXXXXXXXXX**K**	CDR-H2	55	67	K	Oxidation/15.9949	67	0.1	<0.1	99^*^
LC-A	l	XXXXXXXXXXXX**K**GR	CDR-H2	55	69	K	Oxidation/15.9949	67	<0.1	<0.1	
								Sum	0.1	<0.1	
HC-H	nd	NQVSLSCAV**K**		361	370	K	Oxidation/15.9949	370	0.1	<0.1	97^*^
HC-K	s	LTVLSSAST**K**		337	346	K	Oxidation/15.9949	346	3.6	0.1	98
HC-K	l	LTVLSSAST**K**GPSVFPLAPSSK		337	358	K	Oxidation/15.9949	346	3.2	<0.1	
								Sum	6.8	0.1	

Comparison of Hyl levels between wild-type and KO clones of three different TCB mAbs (TCB mAb 1–3) together with the relative reduction in Hyl after PLOD-KO. The modified K is highlighted in bold. If different cleavage versions are quantified together, the single quantities are summed to build a total for the respective locus. The cleaved peptide is marked with an “s” (short) and the non-cleaved version with a “l” (long). Note that not for every locus a peptide with missed cleavage was detected and highlighted in the table with “nd” (not detected); “<0.1” means confirmed detection of modification; yet, no valid concentrations can be generated. ^*^ semi quantitative

In a previous publication, the influence of the Hyl residue on the selectivity of trypsin was reported (Molony et al., 1995; Molony et al., 1998). To consider a possible selectivity loss due to Hyl, the tryptic peptide with one missed cleavage, LTVLSSASTKGPSVFPLAPSSK350, was also evaluated for the presence of a modification. By comparing the XIC intensity scales and calculating the ratios separately for both cleavage species, it was observed that the Hyl ratio is significantly higher (ca. 99%) for the peptides with missed cleavage than the ratio obtained by quantifying the correct cleaved peptides only (ca. 5%) (Figure 1B). The majority of the peptide with missed cleavage is present in the modified form. In contrast to the reported observation by Xie et al. (2016), this result implies that lysine hydroxylation influences the cleavage efficiency of trypsin. For some samples, the unmodified peptide with missed cleavage could not be detected based on MS2 because the precursor intensity was below the MS2 trigger limit. The tryptic peptide with one missed cleavage was only found in its modified form. A comparison of modified and unmodified peptides based on XIC and retention time was not possible. Yet, the MS/MS fragment spectrum of the modified peptide also shows nearly 100% coverage and confirms the modification position for the same lysine K350 located in the HC-K C_H_1 domain (Figures 1C–F).

### Hot spot modification of lysine 350 in crossed C_H_1–VL domain of the knob heavy chain in TCB mAbs

Based on the molecular design of TCB mAbs, one arm contains an N-terminal fused additional Fab binder with similar structural characteristics as the other binder domains (see Figure 1A). By this, the hydroxylation hot spot peptide LTVLSSASTK is present in all three Fab binding domains. As reported earlier by Xie et al. (2016), the hydroxylation modification of the C_H_1 lysine in an analyzed IgG was attributed to an existing XKG consensus sequence, which is a prominent site of modification in several endogenous hydroxylated polypeptides (Xie et al., 2016). Remarkably, only the lysine in the XKG motif located in the C_H_1 heavy chain was detected as the only hydroxylation site although several further XKG motifs exist in the sequence (Xie et al., 2016).

**FIGURE 2:**
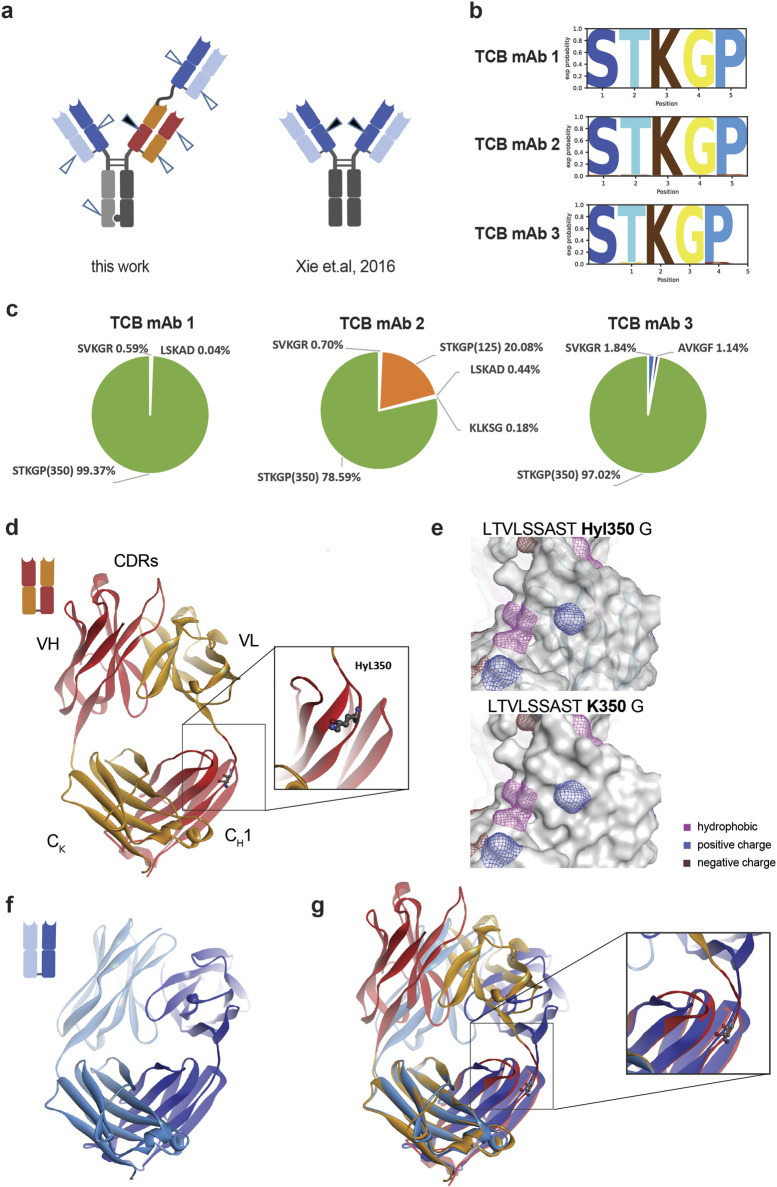
Localization of Hyl modification hot spot and structural consequence in TCB mAbs. **(A)** Several exposed lysine residues were detected by hydroxylation in TCB mAbs (arrowheads). The hot spot (dark arrowhead) with the highest Hyl modification levels is similar to previous observations by Xie et al. (2016) (dark arrowhead). **(B)** Probability analysis of amino acids from positions −2 to +2 identified in hydroxylated peptides identified by LC–MS. **(C)** The percentage distribution of detected Hyl-modified peptides differ for the three analyzed TCB mAbs. TCB mAb 2 is the only molecule where the identical hot spot motif LTVLSSASTK and modified at K125, located in the N-terminal Fab binder. **(D)** Structure of the crossed Fab as represented in red/orange ribbons with Hyl350 (balls and sticks) and the respective domain annotations. The identified hot spot Hyl350 TCB mAbs is located right after the interface of VL to C_H_1 in the HC knob polypeptide chain. **(E)** Predicted influence of hydroxylysine modification on hydrophobicity and positively and negatively charged patches. The prediction is based on *in silico* protonation, energy minimization and protein patch calculation in MOE 2022. **(F)** Structure of trastuzumab Fab domain (pdb code 1n8z), as an exemplary example for a generic IgG Fab, is represented in dark blue and light blue colors for HC and LC, respectively. **(G)** Overlap of a crossed Fab and a generic Fab using the two constant regions C_H_1 and C_K_. The overlap of the structures reveals a difference in the elbow segment and the orientation of the neighbored loop.

Interestingly, the LTVLSSASTK350 peptide located in the crossed HC C_H_1 Fab domain seems to be favored for Hyl modification. Although the hot spot motif is present in all three C_H_1 Fab domains of the three tested TCB mAb molecules, only for TCB mAb 2 a Hyl modification were detected in the further HC-knob LTVLSSASTK125 peptide that did not originate from the crossed HC knob C_H_1 Fab (Figures 1G, 2C). The level of modification is five times lower than the LTVLSSASTK350 peptide located in the crossed C_H_1–VL domain of the knob heavy chain, indicating additional structural requirements for Hyl modification efficiency than solely amino acid sequence identity. For TCB mAb 1 and TCB mAb 3, no Hyl modification of the LTVLSSASTK125 peptide was detected, comprising the same consensus sequence STKGP at lysine125. In contrast to the findings of previous report by Xie et al. (2016), minor yet measurable hydroxylation was detected not only in TCB mAbs within alternative, similar XKG-containing positions but also in different sequence motifs representing XKA and XKS (Figure 2A). The overall propensity of modification in comparison to all hydroxylated peptide sequences detected, however, is dominated by the STKGP sequence motif (Figure 2B).

The prominent hot spot at K350 in the crossed C_H_1–VL domain of the knob heavy chain is near the transition to the chimeric–VL domain, downstream of the intramolecular CDR (Figure 2D). K350 is located within the unstructured C_H_1–VL interface, close to an exposed loop in the C_H_1 domain. We wondered whether the introduction of a hydroxyl group at this position alters the close spatial structure of the C_H_1 domain and physicochemical parameters. Using a molecular prediction approach using artificial lysine protonation, energy minimization, and subsequent protein patch calculation, no substantial differences were observed for surface hydrophobicity, as well as for positive and negative charge patches, indicating either no or only minor effects on overall molecular properties (Figure 2E).

Using a structural model comparison approach, we aimed to decipher the potential structural differences between both LTVLSSASTK motifs, located in the generic N-terminal C_H_1–VH and in the crossed C_H_1–VL domain of the knob heavy chain, respectively, and we also sought to determine whether this analysis could provide an explanation for the observed preferential hydroxylation of K350. For that, the trastuzumab Fab domain (Figure 2F), as an exemplary example of a generic IgG Fab, was overlapped with a crossed Fab domain using the two constant regions C_H_1 and C_K_ for orientation alignment. Structural analysis of the crossed Fab domain by comparing the structural organization of the Fab domain of trastuzumab revealed indeed significant differences in the upstream elbow segment and for a neighboring exposed loop (Figure 2G). The exposition of the elbow segment looks higher in the cross Fab compared to the “regular” Fab, which may be caused by the one amino acid longer elbow sequence for the crossed Fab. In addition, a K350-neighbored loop in C_H_1 adopts a different conformation, which indicates some structural flexibility that may influence eventually the rate of the hydroxylation of lysine 350 by providing favored steric accessibility for potential modifying enzymes.

### PLODs are responsible for lysine hydroxylation in TCB mAbs produced by CHO cell lines

**FIGURE 3:**
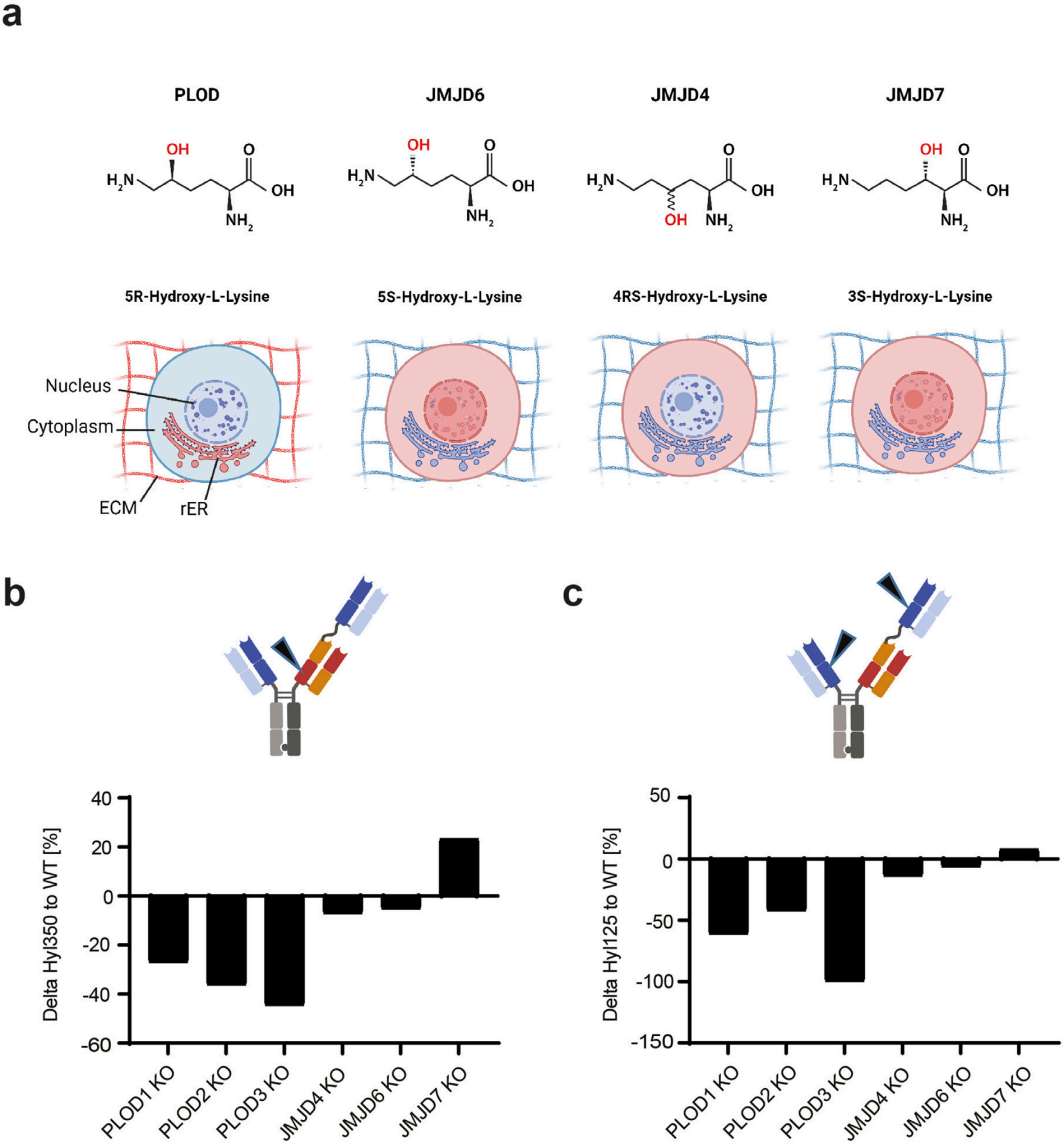
PLODs and not JMDJ hydroxylases are responsible for Hyl modification in TCB mAbs. **(A)** Lysine hydroxylation modification as catalyzed by PLODs, JMJD4, JMJD6, and JMJD7, with respective C-positions in lysine stereospecificity. Different 2OG-dependent hydroxylases, PLOD, JMJD4, JMJD6, and JMJD7 create isobaric yet conformationally different Hyl variants. The enzymatically generated hydroxyl groups are highlighted in red. The cellular localization of the respective lysine hydroxylase, as annotated in UniProt, is shown by red emphasized structures in a mammalian cell (ECM, extracellular matrix; rER, rough endoplasmic reticulum). CRISPR/Cas9 screen of mammalian Lys hydroxylases in TCB mAb 1 expressing production CHO cell line indicate that PLODs are responsible for Hyl modification of TCB mAb 1, as shown by observed peptide Hyl350 **(B)** and Hyl125 **(C)** levels normalized to wild-type controls.

In general, protein hydroxylation can be catalyzed by several 2OG-dependent hydroxylases (Markolovic et al., 2015). In mammalian cells, two enzyme family classes catalyze the post-translational formation of Hyl in proteins: procollagen-lysine, 2-oxoglutarate 5-dioxygenases, comprising PLOD1, PLOD2a (LH2a), PLOD2b (LH2b), and PLOD3 and the Jumonji domain-containing protein family, like JMJD4 and JMJD6 (Markolovic et al., 2015). As reported earlier, the single Hyl modification of an IgG C_H_1 peptide identified by Xie et al. (2016) contains an XKG consensus sequence, which is known to be the site of modification in other hydroxylated proteins such as collagen. Consequently, the authors speculated that the CHO cell-derived homologs of the lysyl hydroxylase complex cause lysine modification (Xie et al., 2016). So far, and to our knowledge, the mechanism and involved enzymatic factors responsible for the Hyl formation of recombinant proteins produced in CHO cells have not been identified. The cellular localization and compartments of enzymatic activity of PLODs and JMJDs are believed to differ by the ER/extracellular matrix and cytoplasm/nucleus/nucleolus, respectively, which suggests that PLODs function as recombinant TCB mAb modifying enzymes (Figure 3A). However, a contribution of JMJDs on TCB mAb hydroxylation by cell lysis induced by stressful bioreactor cultivations and/or cytoplasmic enzyme shedding events cannot be ruled out. In addition, several reports have described the presence of unexpected “classical” cytosolic proteins such as thioredoxin, glutathione S-transferase, and L-lactate dehydrogenase in CHO cell culture extracellular space, which suggested a deeper analysis of potential lysine modulation enzymes (Koterba et al., 2012; Jones et al., 2021). Based on this, we intended to identify specific involved CHO enzymes that cause the detected Hyl modification in TCB mAbs and develop methods to avoid the Hyl presence in the target product proteins.

For this purpose, we employed CRISPR/Cas9 knockout (KO) screen targeting 2OG-dependent lysyl hydroxylases PLOD1, PLOD2, PLOD3, JMJD4, JMJD6, and JMJD7. All these enzymes catalyze lysine hydroxylation, but they differ in the position and/or stereochemistry of the hydroxyl group (Figure 3A). The screen was performed in the CHO genetic background using the TCB mAb 1-expressing CHO production cell line and the CHO reference genome, GCA_003668045.2_CriGri-PICR_genomic. The sgRNAs were designed according to Doench et al. (2016). For each target, three distinct sgRNAs were designed and used for transfection into the TCB mAb 1-expressing CHO production cell line (Supplementary Table S1). Post-recovery, the cells were cultivated for 4 days in batch cultivation, the supernatant was harvested, and the produced TCB mAb 1 was purified and analyzed for Hyl abundance. Unexpectedly, lysine hydroxylation was identified not only in the known hot spot tracer peptide LTVLSSASTK350 but also in the alternative Fab peptide K125. The observed lack of hydroxylation at the K125 position in the initial experiment could potentially be attributed to variations in the cell culture procedures employed (10 day bioreactor fed-batch vs. 4 day shaker batch cell culture process) or inherent discrepancies within the analytical methods utilized. For both analyzed hydroxylated peptides, CRISPR/Cas9 knockout of Chinese hamster PLOD enzymes reduced the lysine hydroxylation level by 27%–99%, with PLOD3 showing the highest effect. For JMJD4 and JMJD6 enzymes, either a small decrease (up to 7%) or even an elevated level of Hyl of 23% by JMJD7 was observed (Figures 3B, C). This approach demonstrated the relevance of PLODs, rather than JMJD enzymes, for TCB mAb lysine hydroxylation. Interestingly, the knockout of JMJD4, JMJD6, or JMJD7 did not have an effect on general cell viability, indicating its essential role in CHO cellular homeostasis (data not shown).

**FIGURE 4:**
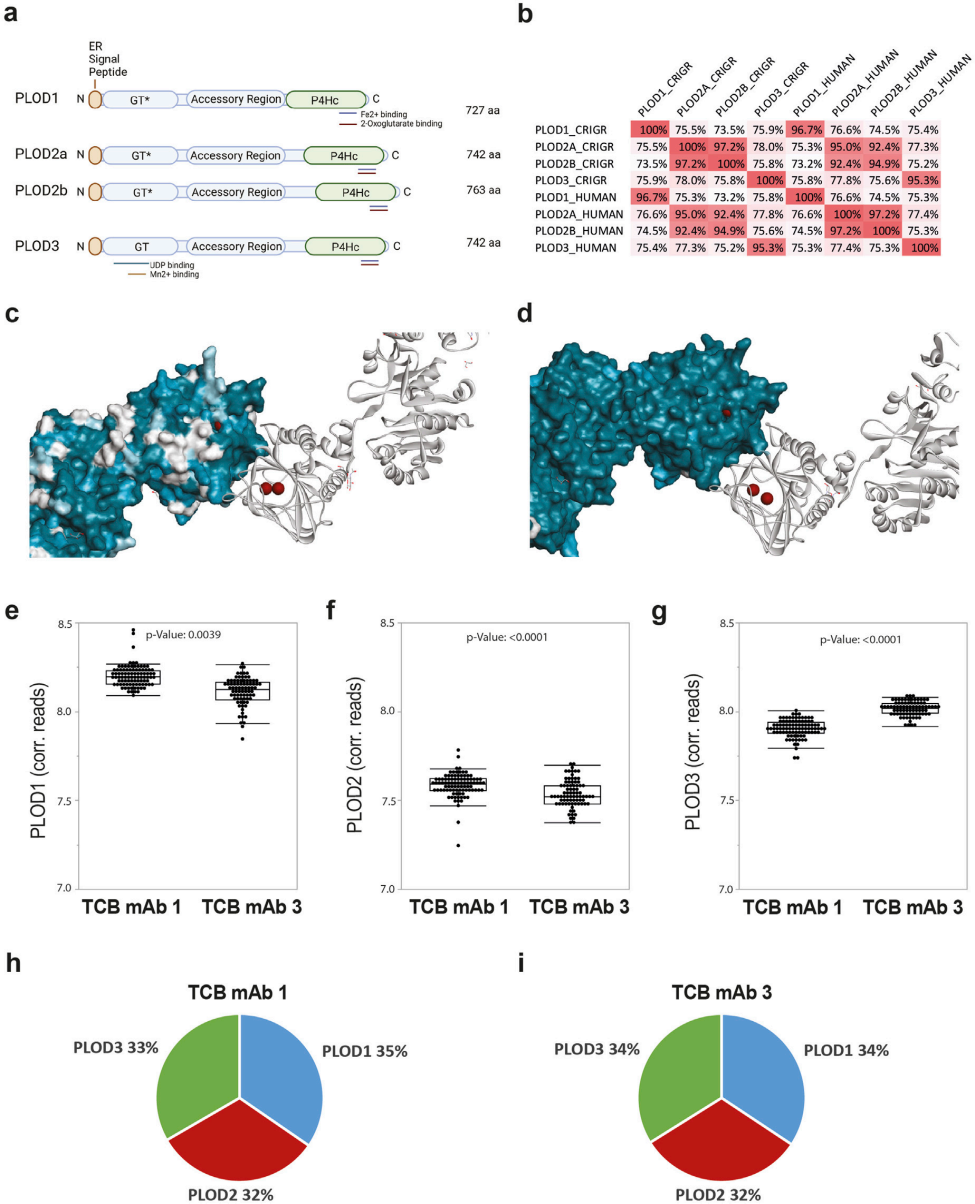
PLODs are expressed by CHO cells and show high homology to human orthologs. **(A)** Schematic representation of Chinese hamster PLOD sequence fishing and the knockout domain organization of Chinese hamster PLOD isoenzymes 1, 2a, 2b, and 3 with the glycotransferase domain (GT, note GT* represents defect activity in PLOD1, PLOD2a, and PLOD2b), accessory domain, and hydroxylase domain (P4Hc), respectively. The respective metal cofactor and substrate binding sites for Mn^2+^, Fe^2+^, UDP, and 2-oxoglutarate, necessary for respective enzymatic activity, are highlighted by colored bars. **(B)** Sequence homology analysis matrix comparing Chinese hamster (CHRGR) to human PLOD isoenzymes. **(C)** Low surface conservation between human PLODs. Structure conservation analysis by mapping PLOD1, PLOD2a, and PLOD2b onto PLOD3 (PDB 6fxt) structure with a focus on the P4Hc subunit domain as the surface. The catalytic Fe^2+^ metal ions are presented in brown. **(D)** High surface conservation between human and Chinese hamster PLOD3. Structure conservation analysis by mapping Chinese hamster PLOD3 onto human PLOD3 (PDB 6fxt) structure with a focus on the P4Hc subunit domain as the surface. The catalytic Fe^2+^ metal ions are presented in brown. Chinese hamster PLOD1 **(E)**, PLOD2 **(F)**, and PLOD3 **(G)** transcript expression levels analyzed by RNAseq analysis in TCB mAb 1 (n = 73) and TCB mAb 3 (n = 84) CHO production clones shown as single events and box plot. The *p*-value was calculated using the Kruskal–Wallis test. Median transcript expression proportion in CHO production clones for TCB mAb 1 **(H)** and TCB mAb3 **(I)**.

For a more detailed analysis of putative further CHO-derived PLOD enzyme homologs to human PLOD enzymes PLOD1, PLOD2A, PLOD2B, and PLOD3, we identified the predicted orthologs of all PLOD genes in the Chinese hamster (s. M&M, Figures 4A, B; Supplementary Figures S6–S9). By this, for all known human PLOD isoenzymes, Chinese hamster homologs could be established. The nucleotide sequence comparison between human and Chinese hamster transcripts and protein sequences shows very high similarity for PLOD1 (96.7%), PLOD2A (95.0%), PLOD2B (94.9%), and PLOD3 (95.3%) (Figure 4B; Supplementary Figures S2A), confirming a high degree of conservation among species and also among other vertebrates (data not shown). Similar to human homologs, PLOD isoenzyme sequence similarity between PLOD1, PLOD2 and PLOD3 is only moderate (for human PLODs: 73.5%–75.9% and for Chinese hamster PLODs: 74.5%–76.6%) and results in significant sequence variations (Figure 4C), while the functional domains are present in all isoforms (Figure 4A). In addition, iron and 2-oxoglutarate cofactors and substrate-binding sites are conserved in Chinese hamster PLOD enzymes, suggesting the same enzymatic activity as in human orthologues (Supplementary Figures S2B, C). An exemplarily additional structural similarity analysis of human and Chinese hamster PLOD3 shows a high degree of molecular similarity, which strongly indicates the same functional activity of PLODs in CHO cell cultures as in humans (Figure 4D).

In a subsequent step, we evaluated the existence and putative differences of Chinese hamster PLOD transcript expression in CHO cells. For this, we first checked the presence of PLOD1, PLOD2, and PLOD3 mRNA transcripts as annotated in an internal RNAseq transcript database containing expression profiles form 157 different CHO production cell lines expressing TCB mAb 1 or TCB mAb 3. Intriguingly, when corrected for gene length, the transcript levels of PLODs in the tested monoclonal CHO production cell lines expressing either TCB mAb 1 or TCB mAb 3 exhibited a significant disparity between the expressed products, with PLOD1 and PLOD3 having an increased transcript expression level compared to PLOD2 (Figures 4E–G). However, each of the PLOD enzymes was transcribed at approximately equivalent yet statistically significant different levels for both tested TCB mAbs, with a slight increase in the abundance of PLOD1 and PLOD2 in TCB mAb 1 and a higher level of PLOD3 in TCB mAb 3-expressing cell lines. However, in the aggregate, each of the PLOD enzymes was transcribed at approximately equivalent levels in CHO cell lines for both tested TCB mAbs, albeit with a slight inclination toward elevated levels of PLOD1 and PLOD3 (Figures 4H, I).

### Gene transcript expression of PLOD isoenzymes is dynamic in CHO cells expressing a TCB mAb, is dependent on the cell culture process, and correlates with the Hyl modification level

Given the significant variation in PLOD isoenzyme transcript levels among individual monoclonal Chinese hamster ovary (CHO) cell lines, we wanted to assess their presence and expression behavior through a 14-day fed-batch fermentation process using Ambr 250 bioreactors, representative for TCB mAb production. We utilized TCB mAb 2 and tested three monoclonal CHO production cell lines stably producing the respective TCB mAb. Additionally, two different process variants, Process A and Process B, were applied, which differ in nutrient concentrations in the feed and the feeding regimen applied.

**FIGURE 5:**
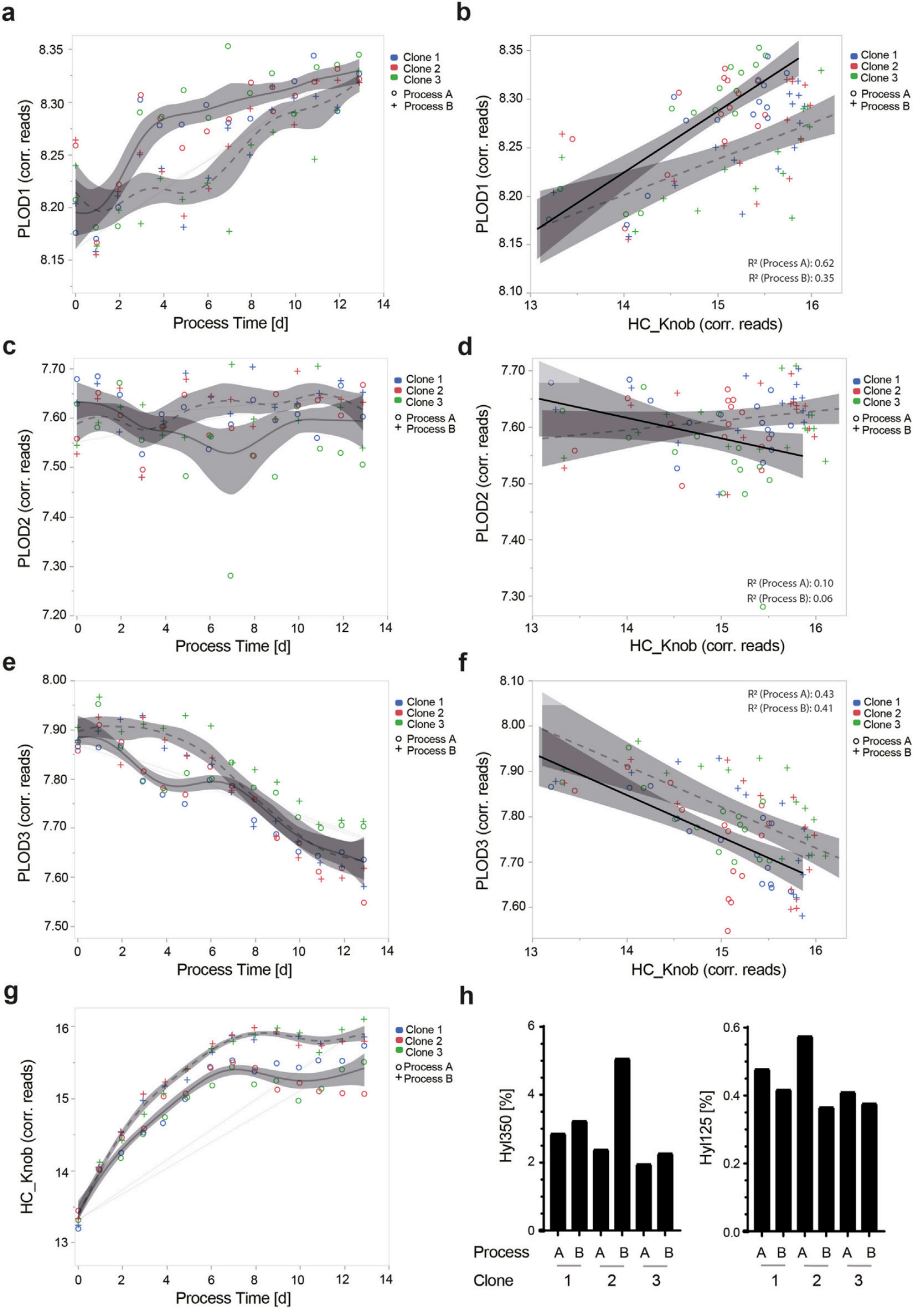
Transcript expression kinetics for PLOD1, PLOD2, and PLOD3 is dynamic in CHO cell culture processes. Gene length-corrected Chinese hamster PLOD1 **(A)**, PLOD2 **(C)**, and PLOD3 **(E)** and HC_knob target protein **(G)** transcript expression kinetics of three different monoclonal production clones, all expressing TCB mAb 2. All clones were cultivated in two different fed-batch fermentation processes (circles: Process A and crosses: Process B). Using linear correlation analysis for PLOD isoenzymes with the HC_knob target protein, the respective endogenous PLODs and ectopic HC_knob gene transcript levels were analyzed **(B, D, F)**. Correlation R^2^ for each process variant is shown in the graphs. The impact of different cell culture process variants A and B using three different CHO expression clones for TCB mAb 2, namely, clone 1, clone 2, and clone 3 on the lysine hydroxylation abundance for Hyl350 and Hyl125 is shown **(H)**.

As previously observed for TCB mAb 1- and TCB mAb 3-expressing clones, the initial transcript expression levels at day 0 of PLOD1, PLOD2, and PLOD3 in TCB mAb 2 clones once again exhibited the same difference in expression levels, with PLOD1 > PLOD3 > PLOD2 (Figures 5A, C, E). Interestingly, the expression kinetics of different PLOD isoenzymes’ transcript levels varied between PLODs and were sensitive to cell culture process variations. While the transcript level for PLOD1 increased over process duration, PLOD2 remained constant, and PLOD3 even decreased (Figures 5A, C, E). The PLOD transcript levels differed between the applied process variants, particularly from days 2 to 6/8 for PLOD1 and PLOD3, correlating with different feeding regime timings of process variants. Interestingly, the initial and final transcript levels were quite similar between process variants A and B (Figures 5A, C, E).

In a subsequent step, we analyzed the ectopic transgene transcript expression level for the HC_knob chain, where the Hyl hotspot motif is encoded, and its expression over the process duration. Remarkably, the transcript level increased until day 8 quite similarly for all three tested CHO production clones, albeit with a clearly increased expression level for Process A. From day 8 to the end of the process, the transcript levels stagnated for both process variants, with a slight difference between the clones (Figure 5G). Overall, the transcript expression levels of the HC_knob chain were approximately twice as high as those of the endogenous Chinese hamster PLOD isoenzymes.

Assuming an approximate transferable expression level from transcript to protein, we analyzed the correlation between the HC_Knob chain and the respective PLOD isoenzymes in CHO production clones. Only PLOD1 and PLOD3 showed a reasonable correlation to HC_knob (PLOD1 for Process A: R^2^ = 0.62 and Process B: R^2^ = 0.35; PLOD3 for Process A: R^2^ = 0.43 and Process B: R^2^ = 0.41), with the process variant being favorable for PLOD1 transcript expression and worse for PLOD3 and *vice versa* (Figures 5B, D, F). Knowing that the Chinese hamster PLOD isoenzymes and HC_Knob transcript levels can be affected by the cell culture process variant, we aimed to analyze the level of TCB mAb 2 Hyl levels. Here, clear, yet opposite, differences in lysine hydroxylation abundances were observed for the hot spot motif at K350 (Hyl abundance for Process B > A) and the motif at K125 (Hyl abundance for Process A > B), with the highest levels detected for clone 2, underscoring the relevance of PLOD enzyme expression level and the importance of testing for cell culture process variants and production clones (Figure 5H).

### Deletion of all three Chinese hamster PLOD enzymes by CRISPR/Cas9 gene knockout is needed to avoid 5R-Hyl formation in TCB mAbs

**FIGURE 6:**
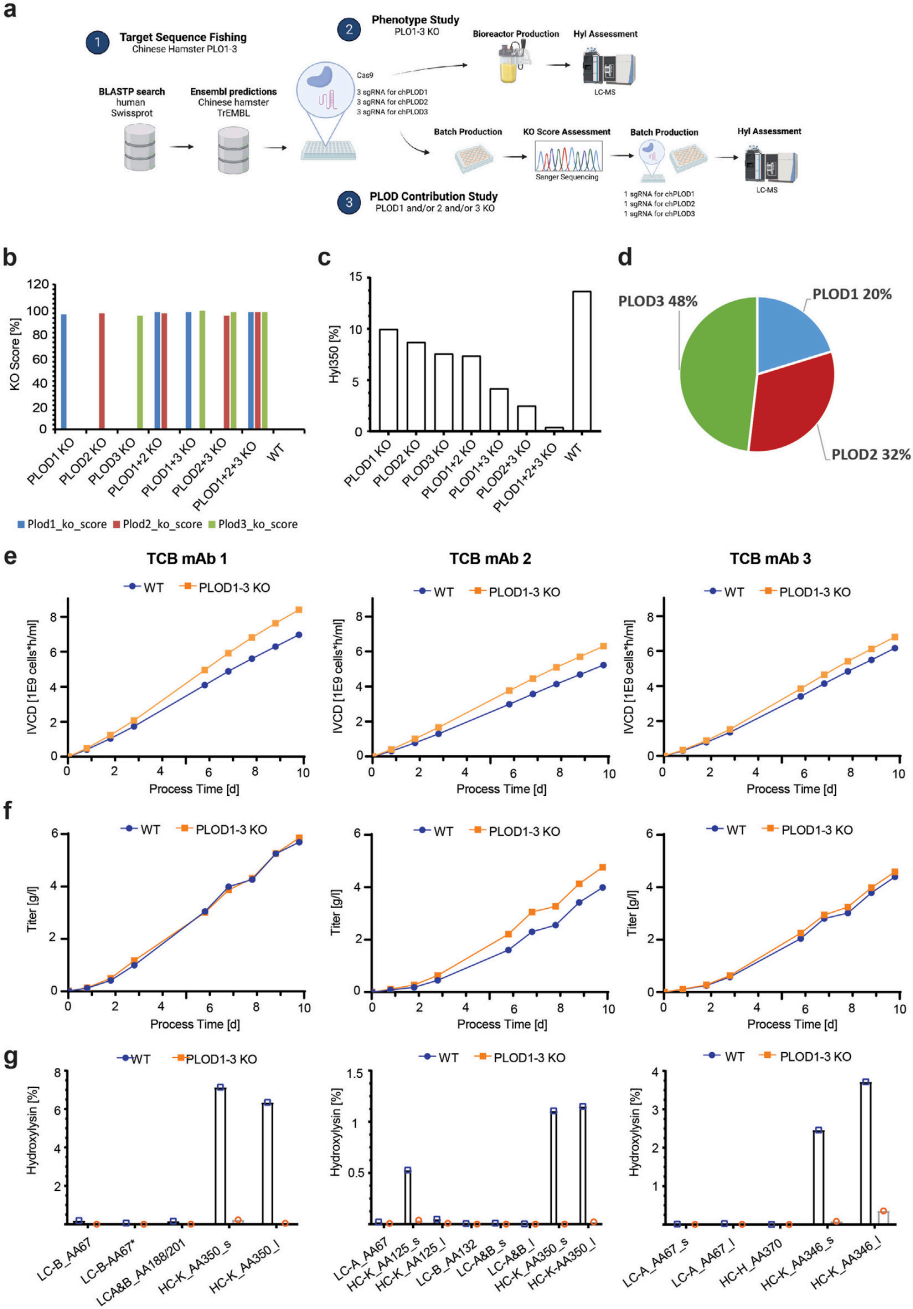
Synergistically gene knockout of Chinese hamster PLOD1, PLOD2, and PLOD3 eliminates Hyl modifications in TCB mAbs. **(A)** Schematic representation of Chinese hamster PLOD sequence fishing and knockout studies. 1) Chinese hamster PLOD gene product orthologs were extracted and predicted by BlastP and Ensembl. sgRNAs were designed for PLOD1, PLOD2, and PLOD3 and used in a subsequent bioreactor “phenotype study” 2), where all chPLODs are deleted using three guide RNAs per *PLOD* gene, and a “PLOD contribution study” and 3), where the most efficient guide RNAs per *PLOD* gene were used. **(B)** The KO score for the most efficient guide RNA per *PLOD* gene used in the “PLOD contribution study” on Hyl modification levels in TCB mAbs. **(C)** Hyl350 levels after CRISPR KO of various combinations of PLOD1–3 in a 4-day batch process using TCB mAb 1-expressing CHO production cell line. **(D)** Contribution of single PLOD on Hyl350 modification based on multi-regression model with “PLOD contribution study” results (see Supplementary Tables for multi regression model results). **(E)** Influence of PLOD1–3 KO on cells in a “phenotype study” during a 10-day fed-batch bioreactor cultivation with TCB mAb 3-expressing CHO production cell line on the integral of viable cell density (IVCD), **(F)** volumetric productivity, and **(G)** hydroxylysine levels across all identified hydroxylysine motives.

The CRISPR/Cas9 KO screen of 2OG-dependent lysyl hydroxylases, the presence of a highly conserved Chinese hamster PLOD homolog, and the TCB mAb modification dominantly observed in the known lysyl hydroxylase consensus sequence XKG strongly suggest that this class of hydroxylases is responsible for the observed lysine hydroxylation modification. We applied a CRISPR/Cas9 gene deletion approach aiming to analyze the Chinese hamster PLOD KO relevance in a production culture and the level of TCB mAbs Hyl modification. In addition, we intended to elucidate which of the three known PLODs, PLOD1, PLOD2, and PLOD3, contributes to the modification and to what extent using two parallel cell culture studies (Figure 6A). Each TCB mAb 1-, TCB mAb 2-, and TCB mAb 3-expressing CHO production cell line was transfected with preselected sgRNAs, targeting PLOD1, PLOD2, and PLOD3 each, as well as the respective Cas9 ribonuclease. Three sgRNAs per target *PLOD* gene were used to ensure most efficient KO effects (Figure 6B, Supplementary Table S2) in the first “Phenotype Study.” The pools were cultivated for 4 weeks after transfection to allow for recovery and expansion of cell biomass needed for subsequent production fermentation evaluation. For this purpose, the wild-type (WT) and KO pools were used in a fed-batch cultivation experiment using a fully controlled Ambr 250 fermentation system. The cell cultures were harvested after 10 days, and purified TCB mAbs were analytically characterized via LC-Ms for Hyl abundance, as described before. The PLOD knockout relevance on Hyl modification formation was evaluated by analyzing the prominent K350 in the tryptic peptides, as described before. A strong reduction in Hyl modification was observed in all noticeable peptides generated form TCB mAbs in PLOD KO compared to WT CHO cell lines (Figure 6F; Table 2). Remarkably, the combined Hyl reduction for the dominant LTVLSSASTK350 peptide that is present in the crossed knob heavy-chain C_H_1 backbone in all three antibodies reached 93% for TCB mAb 1, 98% for TCB mAb 2, and 98% for TCB mAb 3 (Figure 6G).

The knockout of specific target genes may induce pleiotropic effects within the cellular system (Sonnichsen et al., 2005; Liu et al., 2020). In severe instances, these effects could result in undesirable alterations in cellular performance, which may not be compatible with certain cellular protein production cell systems. Analyzing the entire cell culture growth performance, we recognized an integral of viable cell density (IVCD) over time, which is a measure of overall generated cell biomass in a fermentation process (Figure 6E). The expressed TCB mAb titers, however, showed either a slight (for TCB mAb 1 PLOD1–3 KO and TCB mAb 3 PLOD1–3 KO) or substantial increase (for TCB mAb 2 PLOD1–3 KO) compared to WT cell lines (Figure 6F). By this, the overall specific productivity of KO CHO cell lines was slightly lower (for TCB mAb 1 PLOD1–3 KO) or similar (for TCB mAb 1 PLOD1–3 KO and TCB mAb 3 PLOD1–3 KO) to WT cell lines (data not shown). Further in-depth analysis of cell culture key performance indicators, such as cell viability kinetics and nutrient and metabolic byproduct consumption/formation rates, did not show any significant variance in overall metabolic behavior (data not shown). Interestingly, the product quality of the respective TCB mAbs originating from PLOD1–3 KO cell lines assessed by CE-SDS and SEC demonstrated that all three PLOD1–3 KO cell lines had a higher content of the intended TCB mAb main product (Supplementary Figure S4). The HMW1 species, which are annotated as knob-knob homodimer species, showed a lower level in all PLOD1-3 KO cell lines expressing either TCB mAb 1, TCB mAb 2, or TCB mAb 3 compared to wild-type cell lines (Supplementary Figure S4C). In addition, TCB mAb 1, derived from both PLOD1–3 KO and wild-type cell lines, was subjected to molecular charge species analysis using CIEF. A minor but significant reduction in a specific acidic charge species peak was observed in the material from PLOD1–3 KO cell lines compared to wild-type CHO cells (Supplementary Figures S10A).

### Modulation of 5R-Hyl formation in TCB mAbs is dynamic and dependent on iron availability in CHO cell cultures

**FIGURE 7:**
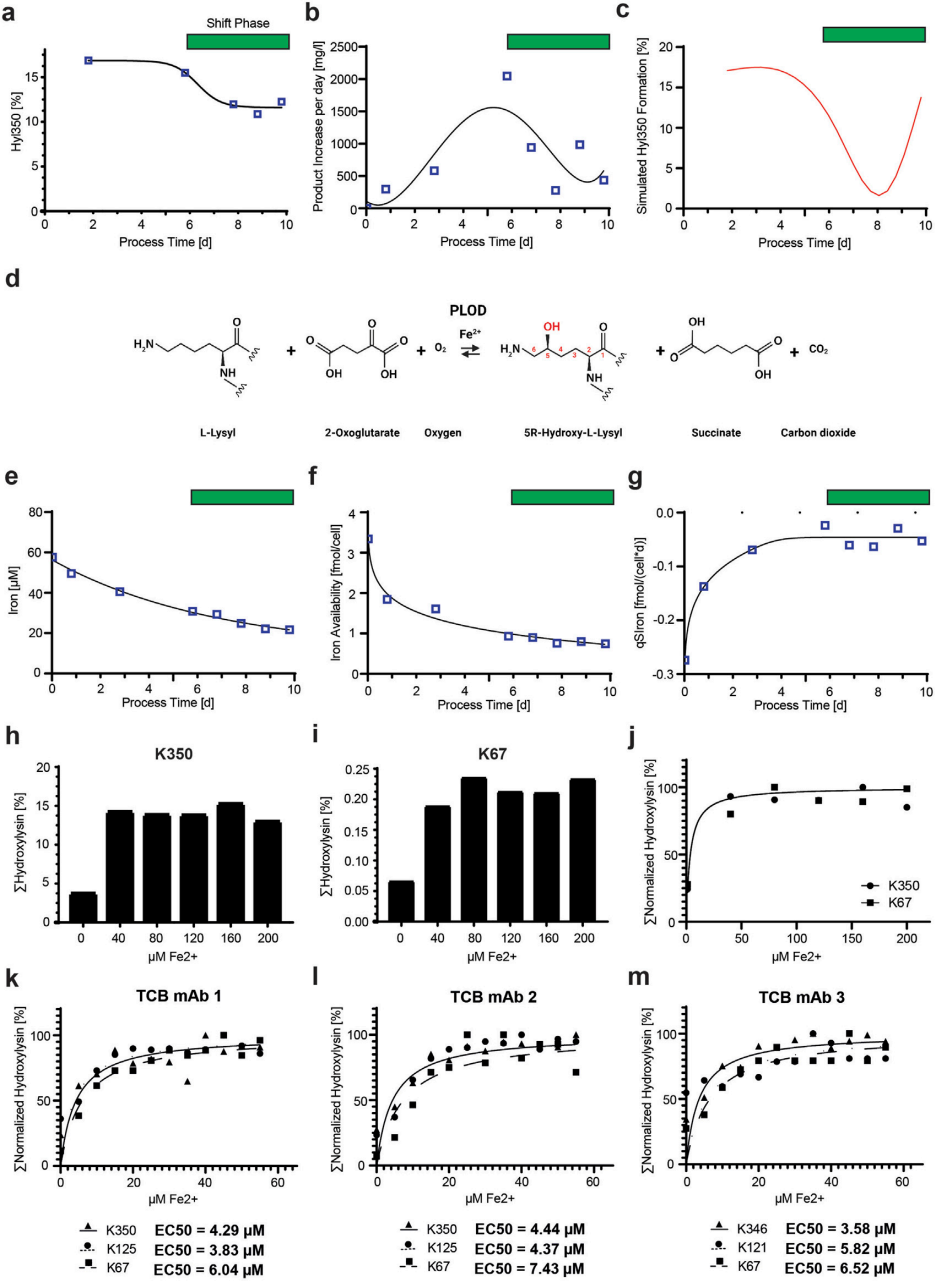
Variability of Hyl modification during cell culture is dependent on iron availability. **(A)** Time course of Hyl350 levels of TCB mAb 1 during a representative fed-batch fermentation process show a decrease in the late process phase. The green bar represents the period of time where the Hyl modification level shifted. **(B)** Kinetic of the delta TCB mAb 1 product increase per day during the fermentation cultivation peaks in the middle of the process. **(C)** Simulated kinetic of %Hyl350 levels newly produced to describe the observed kinetic of product Hyl30 modification in **(A)**. **(D)** Mechanistic description of the enzymatic reactions of PLODs. In proteins, an L-lysyl residue, predominantly located in a Xaa-Lys-Gly consensus sequence, is recognized by the PLOD enzymes and hydroxylated using 2-oxoglutarate and oxygen and the cofactor Fe^2+^ to a 5R-hydroxy-L-lysyl residue, succinate, and carbon dioxide. **(E)** Free iron (Fe^2+^) concentrations measured in the cell-free cell culture supernatant during the fed batch cultivation process. The iron cell density concentrations at a specific process timepoint were used to calculate the iron availability per cell **(F)** and the cell-specific iron uptake rate **(G)**. Both indicate stagnation in trends in the late process phase and suggest a switch in iron metabolism, which is not observed in global iron concentration measurements. Analysis of iron concentration effect on TCB mAb 1 hydroxylation for K350 hot spot motif **(H)** and the lower abundant K67 motif **(I)**. The iron ion activity response curve for Hyl modification stimulation was created by fitting the normalized K350 and K67 hydroxylation levels for the tested iron concentrations using a representative cultivation model **(J)**. Iron ion levels ranging from 0 to 60 µM were tested for all three TCB mAbs in representative cell culture models, and activity response curves for Hyl modification were fitted for three different Hyl-containing peptides using normalized cumulative Hyl levels for each peptide **(K–M)**. EC50 values needed for half-maximum Hyl modification for each peptide were calculated using non-linear least squares fits.

Upon examining the Hyl levels of TCB mAb 1 over the process duration, we observed a trend of decreasing Hyl level in the produced TCB mAb, particularly at the prominent K350 residue located in the intersecting C_H_1–VL domain during the later process phases, beginning from day 6 and continued until harvest on day 10 (Figure 7A). The process productivity at various timepoints was determined by calculating the daily formation of new products, which contributed to the cumulative final product concentration at the time of harvest. A consistent augmentation in the levels of newly formed product was observed until the initiation of the shift phase on day 6, followed by a subsequent decline (Figure 7B). Intriguingly, through the simulation of the time-resolved levels of the newly modified Hyl350 peptide between the sampling days using a sigmoid interpolation function (Figure 7C), we discerned a specific temporal decrease in the levels at the day corresponding to the onset of the global Hyl350 shift on day 6, followed by subsequent growth again (Figure 7B).

As identified in the tested knockout panel, we confirmed that the Hyl modification of TCB mAbs originates from enzymatic PLOD activity. Given that PLODs necessitate Fe^2+^ ions as a cofactor for catalytic activity (Figure 7D), we sought to analyze the available cell culture iron level during the process duration. We observed a clear decrease over time, which became more pronounced when examining the iron availability per cell, where iron levels stagnated at a low level from day 6 onward (Figures 7E, F). This low iron level was also accompanied by a very low steady-state level of cell-specific iron consumption rate *qS*_*Iron*_ from day 6 until the end of the process on day 10 (Figure 7G). Both reduced iron availability and lower cellular iron uptake occur contemporaneously with the shift phase of decreased Hyl350 formation.

In a subsequent experiment, we investigated whether the initial levels of iron in the cell culture media influenced the Hyl modification of TCB mAb motifs. For this, the TCB mAb 1-expressing CHO cell line was cultivated with ferrous ion levels ranging from 0 to 200 µM, and samples of the produced product were subsequently analyzed for Hyl350 abundance. The analysis of the Hyl modification levels of the hot spot motif K350 and the less abundant K67 location, situated near the CDR region of the N-terminal Fab domain, revealed a dose-dependent increase in Hyl modification, reaching saturation with 80 µM iron in the culture (Figures 7H, I). The low level of Hyl modification at 0 µM iron supplementation is likely induced by the residual iron transferred from the precursor culture, which is used to inoculate the experiment. This experiment suggests that levels below 40 µM iron are a rate-limiting factor for Hyl modification in CHO cultures producing TCB mAbs. Furthermore, our objective was to evaluate the concentration of ferrous ions required to achieve the half-maximal effect, as reflected by the observed normalized levels of Hyl350 and Hyl67 in the CHO cell culture experiment. The fitting models suggest that concentrations less than 10 µM Fe^2+^ are already adequate to cause the half-maximum activation of the hydroxylation reactions in the employed CHO cell culture system (Figure 7J).

More in-depth analyses were conducted to examine the iron dependency related to TCB mAbs and different lysine-hydroxylated peptides at iron concentrations below 60 µM. To this end, three distinct CHO cell lines, each expressing one of three different TCB mAbs (TCB mAb 1, TCB mAb 2, or TCB mAb 3), were cultured within a cell culture model representative of production conditions, with Fe^2+^ ion concentrations varying from 0 to 60 µM. For each TCB mAb, three peptides were investigated, encompassing the hydroxylation-sensitive residues K67, K125/121, and K350/K346. The observed levels of hydroxylation were normalized, and the data from the Fe^2+^ ion titration were modeled using a non-linear least squares fitting approach. The half-maximum effective concentration (EC50) for all three TCB mAbs and peptides was determined to be less than 10 µM with a 95% confidence interval (Figures 7K–M). Notably, the hydroxylation of the light-chain residue K67, a motif newly characterized for lysine hydroxylation in this study, exhibited a general trend for a slightly increased requirement for Fe^2+^ ions for hydroxylation, as evidenced by a higher EC50 value. This suggests that a higher concentration of iron is requisite to attain an equivalent level of hydroxylation.

## Discussion

Lysine is one of the most chemically reactive amino acids in biology, and specialized cellular enzymes ensure spatial and temporal regulation of lysine modifications (Abbasov et al., 2021). Lysine hydroxylation is a well-known molecular modification of endogenous proteins, which is often needed for essential downstream regulatory events and structural organizations (Markolovic et al., 2018; Zurlo et al., 2016; Cockman et al., 2022). However, several therapeutic proteins reported in the past contain hydroxylysine instead of lysine in their structure (Molony et al., 1995; Aguilar et al., 2005; Andrews et al., 1984; Xie et al., 2016). In this study, we report for the first time that Chinese hamster PLODs are responsible for 5R-Hyl modification of TCB mAbs produced by CHO production cell lines. For the identification of PLODs as Hyl-forming enzymes in TCB mAbs, a combination of mass spectrometry peptide map analysis and CRISPR/Cas9 KO approach was applied.

The Hyl modification was dominantly located at the C-terminal K350 of the LTVLSSASTK peptide, which is located within the unstructured, crossed C_H_1–VL interface of the heavy knob chain. It was found to a much lesser content at K125 in a similar peptide, LTVLSSASTK, which is located in the N-terminal Fab domain. The hot spot position is similar, but not identical, to the upstream sequence and structural vicinity of the previously described HC101–HC124 motif (XXXXXXXXXWGQGTLVTVSSASTK) (Aguilar et al., 2005) identified in generic IgGs. The reason for the differences observed in TCB mAbs may originate from a unique crossed C_H_1–VL interface that allows for efficient pairing of the intended light chains, which differ for the two identical N-terminal binders and the intra-chain Fab binder (Figure 1A). The structural analysis of the crossed Fab domain revealed favorable accessibility of PLOD enzymes at the K350 position compared to the generic Fab domain in IgGs. These results suggest potential opportunities for further sequence optimization around the LTVLSSASTK350 neighborhood to reduce the observed flexibility as an alternative approach to PLOD enzyme depletion in order to avoid Hyl formation. Interestingly, different PLOD enzymes seem to target favored structural characteristics although the modifying consensus sequence XKG is the same. In procollagens, for example, PLOD2 hydroxylates lysine residues in the telopeptide, and PLOD1 targets lysine residues in the central or α-helical domain (Gilkes et al., 2013a). In contrast, the substrate specificity and favored target motif characteristics for PLOD3 are still unidentified (van der Slot et al., 2003; Takaluoma et al., 2007). Our current example of hot spot-specific Hyl formation in TCB mAbs with multiple XKG motifs supportively suggests that higher-order structural characteristics are important for enzyme target recognition rather than simplified, short consensus sequence definitions.

The modification of lysine residues by 2OG-dependent PLOD hydroxylases is attributed to an XKG consensus sequence (Yamauchi and Sricholpech, 2012). As previously reported by Xie et al. (2016), the generic IgG sequence comprises multiple alternative XKG motifs where a Hyl modification was not detected (Aguilar et al., 2005). TCB mAbs share this characteristic of multiple XKG motifs within their molecule framework; yet small but significant levels of Hyl were detected at alternative XKG sites in the present study. In addition, we detected Hyl at so far unknown XKA and XKS motifs in antibodies. Both motifs are recognized in collagen (Yamauchi and Sricholpech, 2012), suggesting a certain level of flexibility in consensus sequence acceptance of PLOD enzymes in cellular production systems.

The presence of the Hyl moiety in a peptide can abolish tryptic digestion (Markolovic et al., 2018). In contrast to the previous reports by Xie et al. (2016) and Aguilar et al. (2005), the peptide mapping analyses on TCB mAbs showed a clear effect of Hyl on digestion efficiency (Table 2). Approximately half of the prominent, high-abundant peptides with Hyl modification showed a tryptic miscleavage event. Although cleavage levels may be optimized by the applied trypsin activity using higher quantities and/or improved reaction conditions, biological effects, such as Hyl modification influence on trypsin digestion efficiency, may be overlooked via LC–MS. In general, the reason for the additional Hyl spot observations compared to the report by Xie et al. (2016) can be diverse and may be due to a different analytical setup and available detection sensitivity, as well as differences in the structure of the analyzed molecule.

Comparable to humans, we showed for the first time that Chinese hamster ovary cells express all PLOD major isoforms, PLOD1, PLOD2, and PLOD3. The enzyme orthologs are highly conserved between species and own the respective substrate and cofactor-binding sites needed for enzymatic activity. Using a combinatorial CRISPR KO and factor modeling approach, the relevance of different PLOD isoenzymes for TCB mAb lysine hydroxylation was analyzed. Therefore, a dominant role for PLOD3 could be identified, explaining almost half of the observed K350 hydroxylation of the hotspot motif. The reason for the dominant relevance of PLOD3 may be triggered by the PLOD’s unique feature of multiple cellular localization characteristics in the ER and the extracellular environment, where PLOD3 is an important factor in facilitating ECM remodeling (Salo et al., 2006). In our study, we did not analyze the contribution of conditioned cell culture media on TCB mAb Hyl formation by secreted hydroxylases, such as PLOD3, by inhibiting extracellular activity or supplementation with recombinant active enzymes. Consequently, we cannot rule out that the Hyl modification of TCB mAbs in CHO cell cultures also happened as a post-secretion process. Further studies have to be conducted to elucidate this option in CHO cell cultures in detail.

In general, PLODs have been described to play a key role in fibrotic diseases and cancer progression (van der Slot et al., 2003; Mia and Bank, 2015). However, several recent studies on PLODs in cancer suggest that PLOD3 plays an important role in a variety of cancer types, such as in lung and gastric cancer, glioma, and sarcoma (Baek et al., 2018; Deng et al., 2021; Gong et al., 2022). For example, in a most recent study on ovarian cancer, specifically higher PLOD3 expression has been associated with poor prognosis (Li et al., 2020) although all PLOD enzymes are overexpressed in ovarian cancer tissue and cell lines (Guo et al., 2021). Using an RNAi approach, PLOD3 was attributed to specifically regulate gap junction’s functionality by connexin 43 gene expression. Therefore, the authors concluded that the different PLOD isoenzymes possess non-overlapping functionality in ovarian cancer with unexpected impact on gene regulation networks in addition to their direct enzymatic roles in hydroxylation and glycosylation of target proteins (Guo et al., 2021).

In comparison to the distinct roles of PLODs in cancer, our data showed that all three PLOD enzymes must be depleted from the cellular system to quantitatively avoid lysine hydroxylation for TCB mAbs, as suggested by the “PLOD contribution” study on TCB mAb 1 (Figures 6C, D). Interestingly, no unintended cell culture performance decrease was detected by a concomitant loss of all Chinese hamster PLOD isoenzymes, chPLOD1, chPLOD2, and chPLOD3, using a triple knockout CRISPR/Cas9 approach. In contrast, the three different production cell lines modified by PLOD1–3 KO showed a significantly increased cell growth performance as well as a minor but obvious improvement in final product concentration and intended main product levels. These results were not expected, especially not for observed larger biomass formation since PLODs have been attributed to cancer progression, metastasis, and cancer cell proliferation (Baek et al., 2018; Song et al., 2017). For example, decreasing PLOD1 expression impairs the proliferation and colony-formation capacity of A549 lung cancer cells by the activation of E2F transcription factor 1 (E2F1) (Li et al., 2021). Although immortalized CHO cells also share common hallmarks of cancer cells [see review by Wurm and Wurm (2021)], the biological relevance of PLODs may be different from reported cancer cell observations due to extensive evolution as highly adopted production cell lines for growing in suspension cultures (Wurm, 2004). Our study cannot exclude the unlikely event that the observed differences in cell culture performance between wild-type and PLOD1–3 knockout cell lines may, in addition, be influenced by indirect effects. PLOD enzymes are known to interact with various factors, leading to multifunctional biological effects, as provided in Table 3. Past research has identified distinct interaction partners for PLOD1, PLOD2, and PLOD3, suggesting that they have unique biological roles (Heard et al., 2016; Scietti et al., 2018; Duran et al., 2017; Gjaltema et al., 2016; Liefhebber et al., 2010). However, the regulatory networks involving PLOD enzymes are not fully understood, particularly in the context of cellular biologics production systems like CHO cells. For the analysis of regulatory mechanisms that differentially influence recombinant protein expression in wild-type and PLOD knockout CHO cells, further comprehensive comparative studies on the system level are needed. These studies should include transcriptomic, proteomic, and metabolomic profiling to provide a holistic understanding of cellular changes and their impact on biological production.

**TABLE 3:** Lysine hydroxylase enzymes.

Enzyme *Splice variant*	EC numbering	Lysine modification	Domain	Enzymatic activity	Cofactor	Consensus sequence	Organization	Localization	Interaction partners (Heard et al., 2016; Scietti et al., 2018; Duran et al., 2017; Gjaltema et al., 2016; and Liefhebber et al., 2010)
PLOD1	1.14.11.4	(5R)-5-hydroxy-L-lysine	GT_LH P4Hc	Lysyl hydroxylase (LH) (N-terminal)	Fe^2+^ and ascorbate	-Xaa-Lys-Gly-	Homodimer^a^	rER	SC65, P3H3, P3H4^b^, and CYPB
PLOD2 2a/b	1.14.11.4	(5R)-5-hydroxy-L-lysine	GT_LH P4Hc	Lysyl hydroxylase (LH)	Fe^2+^ and ascorbate	-Xaa-Lys-Gly-	Homodimer	rER ECM	HSP47, FKBP65, and BiP
PLOD3	1.14.11.4 2.4.1.50	(5R)-5-hydroxy-L-lysine	GT_LH Glyco_tranf_2_3 P4Hc	Lysyl hydroxylase (LH) Galactosyltransferase (GT)	Fe^2+^ and ascorbate Mn^2+^	-Xaa-Lys-Gly- -Xaa-Hyl-Gly-	Homodimer	rER ECM	GLT25D1/2
JMJD4 2 splice variants	1.14.11.-	(4RS)-5-hydroxy-L-lysine	JmjC	Lysyl hydroxylase (LH)	Fe^2+^	Not known	Homodimer	Cytoplasm	DRG1 and DRG2
JMJD6	1.14.11.4	(5S)-5-hydroxy-L-lysine	JmjC	Lysyl hydroxylase (LH)	Fe^2+^	Not known	Homo oligomerizes	Cytoplasm Nucleus Nucleolus	LUC7L2, LUC7L3, U2AF2/U2AF65, CDK9, CCNT1, and BRD4
JMJD7	1.14.11.63	(3S)-5-hydroxy-L-lysine	JmjC NLS	Lysyl hydroxylase (LH)	Fe^2+^	Not known	Homo oligomerizes	Cytoplasm Nucleus	LUC7L2, LUC7L3, U2AF2/U2AF65, CDK9, CCNT1, and BRD4

^a^ By similarity; P4Hc, prolyl 4-hydroxylase alpha subunit homologs; GT_LH, catalytic glycosyltransferase (GT) domain found in the lysyl hydroxylase (LH) family–including UDP and Mn binding sites; Glyco_tranf_2_3, glycosyltransferase-like family 2; JMJD, Jumonji domain-containing protein.

^b^ NLS, nuclear localization signal motifs. Interaction partners are extracted from the literature and UniProt database (https://www.uniprot.org/).

Mammalian cells harbor two primary classes of 2OG-dependent lysyl hydroxylases: PLODs and JMJDs. These mammalian lysyl hydroxylases exhibit differences in their cellular localization, chiral activity, and the formation of structurally distinct enantiomer Hyl products, specifically 5R-Hyl (catalyzed by PLODs), 5S-Hyl (catalyzed by JMJD6), 4RS-Hyl (catalyzed by JMJD4), or 3S-Hyl (catalyzed by JMJD7). Despite the fact that these hydroxylases have been reported to localize in different compartments, the possibility of unexpected dysregulated intracellular trafficking (Targa et al., 2023), enzyme mislocalization (Wang and Li, 2014), and/or enzyme shedding from disrupted cells (Koterba et al., 2012) contributing to or causing the observed Hyl modification in TCB mAbs cannot be excluded. Our initial CRISPR/Cas9 screen strongly suggested that PLODs, not JMJD lysine hydroxylases, are responsible for TCB mAb modification. Although knockout screens using CRISPR/Cas9 are an established methodology for identifying enzymatic factors relevant to a specific phenotype, the effect can also be indirect, as the deleted target gene product can act upstream of the actual modifier (Bock et al., 2022). Orthogonal methods such as gene overexpression (Bauer et al., 2022) or analytical approaches (Markolovic et al., 2018) are essential tools to validate the genes identified by CRISPR/KO screens. Given that JMJDs generate Hyl enantiomers, identifying the respective Hyl enantiomer would further substantiate that a specific hydroxylase class is responsible for the observed Hyl modification in CHO-produced TCB mAbs. For this approach, Hyl-containing TCB mAb 3 would need to be digested by trypsin, and a tracer peptide like LTVLSSASTK/Hyl would need to be selectively collected. Subsequent digestion of the isolated peptide with carboxypeptidase Y and derivatization of the isolated amino acids by 6-aminoquinolyl-N-hydroxysuccinimidyl carbamate (AQC) would allow for chiral characterization of the respective Hyl stereoisometry and variant (Markolovic et al., 2018). Utilizing this methodology, the approximate +16 Da modification at the identified lysine residues in CHO-produced TCB mAbs could be definitively characterized as 5R-Hyl (Supplementary Figure S5). This strongly substantiates that PLODs, rather than any other 2OG-dependent lysyl hydroxylase, are accountable for the observed hydroxylation of lysine residues.

### Process-induced variability of Hyl modification

All three analyzed TCB mAbs in this study exhibited Hyl modification at different levels, especially at the hot spot motif LTVLSSASTK350. Interestingly, the levels were different although the production cell cultivation process was apparently similar for all three TCB mAbs. Furthermore, the rate of Hyl modification at the K350 residue for TCB mAbs diminishes over the duration of the cell culture process, as exemplified by TCB mAb 3.

Most therapeutic proteins, such as TCB mAbs and generic IgGs, use dynamic cell cultivation processes and high-performance CHO production cell lines [see review by Kim et al. (2012)]. Although these CHO production cell lines originate from the same CHO ancestor cell line and related CHO host cell sublineages, each final production clone demonstrates a more or less unique metabolic profile (Popp et al., 2016). As a consequence, the availability of specific substrates and cofactors needed for biocatalytic processes is tightly linked to cell-specific consumption rates of small molecules present in cell culture. In the conducted research, we demonstrated that iron, acting as a cofactor for enzymatic hydroxylation activity in PLODs, was empirically validated to modulate the formation level of Hyl in TCB mAbs produced by CHO cells *in vitro*. Our study observed a dynamic alteration in the per-cell iron availability and consumption rate, which diminished during the later stages of the cell cultivation process. This fluctuating iron availability for cells could be attributed to various factors, such as different cell culture process phases, variations in cell culture media compound concentrations, impurities from other media and process components, and/or the overall cellular iron metabolism. The observed reduction in cofactor bioavailability further substantiates this hypothesis. Similar to iron, the availability of other requisite factors in cell culture for PLOD activity, such as 2OG, may also have a significant influence. Additional investigations are imperative to decode the presence and temporal regulation of 2OG in CHO cell culture processes.

The transcriptomic analysis of Chinese hamster PLOD1, PLOD2, and PLOD3 unveiled a significant disparity in expression levels among PLOD isoenzymes, with the hierarchy being PLOD1 and PLOD3 surpassing PLOD2 (Figures 4E–G). Fluctuations in PLOD transcript expression levels, originated by external triggers, have also been documented in other cellular models and primary tissues, suggesting varying biological roles and importance (Qi et al., 2021). In addition, transcript expression levels vary among different monoclonal CHO clones, despite all expressing the identical TCB mAb molecule. This suggests that the translation of transcript levels into enzymatic activity may explain the observed variations in Hyl modification across TCB mAb 1, TCB mAb 2, and TCB mAb 3. The observed variation could potentially be amplified as the expressions of the *PLOD1* and *PLOD2* genes have been demonstrated to be regulated by hypoxic conditions (Gilkes et al., 2013a; Gilkes et al., 2013b; Wang et al., 2021). Hypoxic scenarios can occur in bioprocesses due to transitions into different scales and alterations in system aeration configurations (Fu et al., 2014).

The application of various production process variants, which include different media and feeding strategies, and/or physicochemical parameters such as cell culture pH and temperature set points, has been documented to influence the quality of the therapeutic product molecule (Ehret et al., 2019; Lee et al., 2021; Reinhart et al., 2015). Specifically, the use of an optimized pH and temperature process regime leads to a reduction in the level of a therapeutic interleukin-2 variant (IL2v) cytokine–mAb fusion protein (Schneider et al., 2019). In our study, we evaluated the effects of using different production processes and cell lines on the production of TCB mAb 2. We found that both the production processes and the cell lines may influence the regulation of PLOD transcript levels. Intriguingly, the variation in transcript levels appears to be cell line-dependent, underscoring the significance of representative cell line screening procedures and appropriate readouts. This is particularly critical in the context of product quality evaluation, which should also encompass early, comprehensive, and extended characterization studies utilizing mass spectrometry. In addition, the analysis of ectopic product transcript expression is also recommended since it aids in predicting the performance of cell line production (Dorai et al., 2006; Bhoskar et al., 2013). Beyond productivity prediction, the expression of the heavy-chain (HC) knob gene transcript, encompassing the hotspot motif LTVLSSASTK350, was observed to increase in a pattern similar to the *PLOD1* gene transcript in our investigation.

### Consequence of Hyl modification for biotherapeutic TCB mAbs

Therapeutic pharmaceuticals often display microheterogeneity, which necessitates an in-depth characterization to determine their impact on drug efficacy and safety and the establishment of analytical control strategies to maintain consistent product quality (Scietti et al., 2018). The examination of Hyl modification sites and levels calls for intricate and time-consuming mass spectrometric methodologies. Multiple Hyl modifications were identified in TCB mAbs in this study, but apart from the hot spot motif LTVLSSASTK350, the levels in other motifs are remarkably low, less than 1%. Given the extremely low levels, especially in the CDRs, the probability that Hyl modifications in TCB mAbs affect binding efficacy to intended targets or functionality is quite low; yet, additional investigations need to be undertaken to substantiate this hypothesis. Since both clonal variations and process conditions can influence the level of Hyl modification, it is recommended to incorporate Hyl analysis early in the process to avoid selecting clones with significant Hyl modifications. Generally, Hyl modification is a common biological phenomenon, and unexpected reactions, such as immunogenicity, are not typically anticipated.

The hotspot motif LTVLSSASTK350 is situated within the chimeric C_H_1–VL domain. Structural analysis conducted in our study indicated a conformational divergence in the elbow segment and the neighboring loop when compared to the generic IgG Fab domain, as found in the N-terminal binders. Beyond the conventional methodologies for the capture and purification of mAbs that target the Fc region using agents like protein A, (Hober et al., 2007), there are alternative binders that target the C_H_1 domain (Nesspor et al., 2020). These commercially available C_H_1 binders can be utilized for affinity capture in both analytical and preparative chromatography. There is a potential risk that Hyl modification of the exposed C_H_1 binding site could influence this binding although its overall contribution might be limited, considering that TCB mAbs contain one chimeric C_H_1-VL cross-Fab segment and two generic C_H_1 domains.

The analysis of PLOD1–3 KO cell lines expressing TCB mAb 1 revealed a diminished peak of a specific acidic molecular charge species compared to wild-type CHO cells. Hydroxylysine exhibits distinct dissociation constants relative to lysine (Klemperer et al., 1942). Klemperer et al. (1942) suggested that the presence of a hydroxyl group in hydroxylysine reduces its basicity compared to lysine, which could partly explain the observed decrease in acidic species in a material containing >12% hydroxylysine versus less than 1% in TCB mAb 1 (Supplementary Figures S10B, C). However, the unlikely case that additional minor other differences in the product quality profile may contribute to the observed differences in the charge pattern, or if both effects act synergistically to modulate the molecular charge pattern, yet in a small amount, cannot be excluded.

The crosslinking and stabilization of collagen fibrils in the extracellular space have been shown to be influenced by both enzymatic and non-enzymatic processes. Enzymatic processes are mediated by PLODs and lysyl oxidases, which trigger lysine modifications, whereas non-enzymatic processes are facilitated by advanced glycation end products (AGEs) and riboflavin/UV-mediated reactions (Gelse et al., 2003; McKay et al., 2019). PLOD3 is of particular importance due to its additional glycotransferase activity, which produces specific glucosyl-galactosyl-hydroxylysine (Glc-Gal-Hyl) AGE modifications necessary for the non-enzymatic crosslinking of collagen fibrils. Despite not detecting any further AGEs in the respective Hyl-modified motifs in our studies, we cannot rule out the possibility that minor amounts of the intended TCB mAb may be modified. This could lead to the product being cross-linked by a process originating from Hyl, as indicated by the observed increase in TCB mAb monomer content and decreasing trends in high molecular weight (HMW) fractions. Moreover, forced stability testing of TCB mAb 1 and TCB mAb 2 in phosphate-buffered saline (PBS) at 40°C for 4 weeks revealed a minor increase in HMW species although the overall Hyl content remained constant (Supplementary Figure S1). This suggests that non-enzymatic product crosslinking, potentially originating from micro heterogeneities such as hydroxylation modifications, could occur. These observations warrant further investigation in subsequent studies.

### Conclusion

In this study, we document for the first time the occurrence of Hyl modification in TCB mAbs produced by Chinese hamster ovary (CHO) production cell lines. The modification transpires at several XKG positions and, to a lesser extent, in alternative positions, underscoring the importance of comprehensive mass spectrometric characterization efforts to identify post-translational microheterogeneities. The prominent Hyl350 modification is situated in the artificial C_H_1–VL cross Fab interface. The crossing of the chains induces an altered structural situation and a more flexible, exposed hot spot motif LTVLSSASTK350 compared to generic C_H_1–VH interfaces in IgG Fab domains. This may account for the increased Hyl modification compared to the generic LTVLSSASTK125 motif. Chinese hamster PLOD enzymes are forcefully suggested as mediators of the Hyl modification in TCB mAbs. A synergistic knockout of all three isoenzymes, PLOD1, PLOD2, and PLOD3, is necessary to prevent Hyl formation in TCB mAbs compared to single knockouts. Interestingly, the cell culture and productivity performance remain unaffected or even improved compared to the WT control. Overall, Hyl modification does not induce functional or safety issues and has been classified as a monitoring post-translational modification. Further research is required to definitively understand the role of PLOD enzymes in influencing other quality attributes besides lysine hydroxylation of TCB mAbs. Acquiring knowledge on the role of PLOD enzymes may contribute to enhancing the robustness and consistency of biologics production processes through the development and integration of targeted control strategies.

We propose an approach to circumvent Hyl modification in TCB mAbs by eliminating the respective enzymatic factors and/or activity through alternative strategies. This strategy can likely be applied to other products produced by CHO cell lines to avoid labor-intensive characterization activities in the development of protein-based therapeutics.

## Materials and methods

### Cell culture

All cell lines were created using a previously generated CHO host cell line (International patent publication number WO 2019/126634 A2). CHO cells were cultured in a chemically, protein-free medium in 125–500 mL shake flask vessels at 150 rpm, 37°C, 80% rH, and 5% CO_2_. Cells were passaged at a seeding density of 3–6 × 10^5^ cells/mL every 3–4 days. Pools of cells that stably express TCB mAb molecules were generated as described by Carver et al. (2020). In brief, expression plasmids were transfected into CHO cells via MaxCyte STx electroporation (MaxCyte Inc., Rockville, Maryland, United States). Transfected cells were then selected, and the expression of TCB mAb was confirmed by flow cytometry via human IgG staining using a BD FACSCanto II Flow Cytometer (BD, Eysins, Switzerland). CHO clones were selected after single-cell cloning by limited dilution, titer and binder validation by ELISA, and the evaluation of cell growth and productivity performance in fed-batch production assays using Ambr 250 bioreactors (Sartorius AG, Göttingen, Germany).

### Fed-batch production assay

Fed-batch production cultures were performed in 24-deep well plates, shake flasks, or Ambr 250 bioreactors (Sartorius AG, Göttingen, Germany) using proprietary chemically defined production media. Cells were seeded as indicated between 2 and 15 × 10^6^ cells/mL on day 0 of the production stage after adaptation to production media during two passages. Cultures received daily proprietary feed medium after day 3 and additional feed bolus on days 3 and 7, with an optional bolus on day 10. Cells were cultivated for 14 days. Production in the Ambr 250 system was operated at set points of 35°C, DO 30%, pH 7.0, and an agitation rate of 1,300 rpm, with a shift to 1,600 rpm on day 3.5.

### Off-line sample analysis

Process parameters were analyzed using Osmomat Auto (Gonotec GmbH, Berlin, Germany) for the measurement of osmolality and a Cedex Bio HT Analyzer (Roche Diagnostics GmbH, Mannheim, Germany) for the measurement of product and selected metabolite concentrations, including iron. Total cell count, viable cell concentration, and average cell diameter were measured using the Cedex HiRes Analyzer (Roche Diagnostics GmbH, Mannheim, Germany). Integrated viable cell density (IVCD) and specific productivity rates for each condition are calculated as indicated in Equation 1 and Equations 2–4, respectively

$$IVCD_{n}=IVCD_{n-1}+\frac{VCD_{n}+VCD_{n-1}}{2}*\left(t_{n}-t_{n-1}\right).$$ (1)

The cell-specific productivity *qP* and iron consumption rate *qS*_*Iron*_ are calculated as follows:

$$\left(\text{dS}\text{or}\mathrm{dP}\right)/\text{dt}=\left(\text{qS}\text{or}\text{qP}\right)*X$$ (2)

$$\text{qP}=\frac{1}{X}*\frac{P_{i}-P_{i-1}}{t_{i}-t_{i-1}},$$ (3)

$${\text{qS}}_{Iron}=\frac{1}{X}*\frac{S_{i}-S_{i-1}}{t_{i}-t_{i-1}}.$$ (4)

Negative and positive values for *qS* and *qP* represent consumption and production of a compound, respectively.

### Chinese hamster PLOD sequence database extraction

The ortholog PLOD protein sequences from Chinese hamsters were identified by BlastP searches of the human Swiss-Prot PLOD entries (PLOD1_HUMAN, PLOD2_HUMAN isoform1 “A” and 2 “B,” and PLOD3_HUMAN) against UniProt and RefSeq. The identified PLOD entries reflect the top hits in TrEMBL for Chinese Hamster and are all based on predictions from Ensembl (PLOD1: A0A8C2QN41, PLOD2A: A0A8C2MXC8, and PLOD2B: A0A8C2N5H0, PLOD3: manually assembled based on A0A8C2MCH2 and A0A3L7HLU9). All these hits are confirmed by RefSeq (except PLOD3 AAs #1-41, which is solely based on TrEMBL).

### Analysis of PLOD transcript expression levels in CHO cells by RNAseq

The library prep needed for RNAseq sample measurements was done using a proprietary rRNA depletion protocol. RNA sequencing was performed on a NovaSeq 6000 System (Illumina Inc.) using read mode SE100 for TCB mAb 3 and PE100 for TCB mAb 1 and TCB mAb 2.

For RNAseq data preprocessing, the quality of the raw FASTQ files was evaluated using FastQC software (version 0.11.9). For the adapter’s removal (ILLUMINACLIP: TruSeq3-PE.fa:2:30:10), Trimmomatic software (version 0.39) was used. Sequences for the transgene and the reference genome were mapped and further analyzed separately. Reads were aligned to the CDS sequences of *PLOD1*, *PLOD2*, and *PLOD3* genes using HISAT2 software (version 2.2.1). Feature quantification was performed using HTSeq (version 0.13.5). The final reads were normalized to the library size and library composition using edgeR’s (version 3.38) (Robinson et al., 2010) trimmed mean of M-value method (TMM).

### Structural modeling analysis

A homology model of the cross Fab was constructed utilizing MoFvAb/IgNORANT, with the incorporation of Hyl achieved through MOE 2022. Structures were protonated at pH 7.4, and energy was minimized using MOE 2022. The trastuzumab Fab domain (PDB code 1n8z) served as a representative example of a generic IgG Fab.

For the evaluation of physicochemical parameters, such as hydrophobicity and the presence of positively or negatively charged patches, both the non-modified and Hyl-modified SSASTK360 motifs of C_H_1–VL were analyzed using MOE 2022.

Surface conservation analysis was conducted for PLODs, as revealed by structure conservation analysis. This involved homology model building of PLOD1, PLOD2a, and PLOD2b using AlphaFold 2, according to Jumper et al. (2021), onto the PLOD3 structure (PDB code 6fxt).

### Generation of pooled single-gene KOs and KO confirmation

Targets were rationally chosen, and guides were designed using Geneious Prime software (version 2020.2.4., off-target library: CHO Reference Genome GCA_003668045.1_CriGri-PICR_genomic). All sgRNA sequences and verification primers are listed in Supplementary Table S1. For knockout (KO) introduction, three recombinant CHO production clones producing TCB mAb 1, 2, and 3 were transfected using ribonucleoprotein complexes comprising gene-targeting sgRNA and Cas9 protein using the MaxCyte STx Electroporation System (MaxCyte, Inc.) or the Lonza 96-well Shuttle System (Lonza Group Ltd.). Guide RNA–Cas9 ribonucleoprotein (RNP) complexes were prepared by mixing 1–3 sgRNA (40 pmol per sgRNA) with an equimolar amount of Cas9 protein (TrueCut Cas9 Protein v2, Thermo Fisher Scientific Inc.), followed by incubation at room temperature (RT) for 20 min. Cells were washed in PBS (300g, 5 min) and resuspended in the respective electroporation buffer (SF Cell Line 4D-Nucleofector™ X Kit, Lonza Group Ltd. or MaxCyte EP buffer, MaxCyte Inc.).

Genomic DNA was extracted using QuickExtract™ DNA Extraction Solution (Lucigen) according to the manufacturer’s instructions after cells had recovered from transfection (6–8 days post-transfection). PCR (98°C for 30 s; 35 times: 98°C for 5 s, 60°C for 20 s, 72°C for 90 s, and 72°C for 90 s) was performed with the Q5 Polymerase 2x Master Mix (New England Biolabs, Inc.). Amplicons were purified using the QIAquick^®^ 96 PCR Purification Kit (QIAGEN). Sanger sequencing was performed by Microsynth AG (Balgach, Switzerland). The PCR products produced were analyzed via electrophoresis on 2% agarose.

KO scores were assessed by the offline version of ICE software for analyzing Sanger sequencing data (available at https://github.com/synthego-open/ice).

### TCB mAb aggregate, fragment, and charge variant analytics

Supernatants were clarified (1,000 g, 30 min, 4°C centrifugation, and 1.2 μm filtration; AcroPrep 96-well Filter Plates, Pall Corporation). Chromatography of analytical protein A was performed via UHPLC with UV detection (Dionex UltiMate 3000 UHPLC fitted with POROS™ A 20 µm Column, Thermo Fisher Scientific Inc.).

Antibody integrity was analyzed after protein A affinity chromatography (PreDictor RoboColumn MabSelect SuRe, Cytiva) and normalization with protein quantitation using UV measurement (NanoQuant Infinite M200, Tecan). The percentage of correctly assembled antibodies (Main-Peak) was assessed by CE-SDS (HT Antibody Analysis 200 assay on the LabChip GXII system, PerkinElmer) under non-reducing conditions by relative quantification of the expected protein size to total protein content.

Size exclusion chromatography (SEC) for the determination of the aggregation and oligomeric state of recombinant immunoglobulins was performed via HPLC chromatography. In brief, protein A purified product was applied to a TSKgel QC-PAK GFC 300 Column (Tosoh Bioscience) or a Tosoh TSKgel UP-SW3000 Column in 250 mM of KCl and 200 mM of K_2_HPO_4_/KH_2_PO_4_ buffer (pH 6.2) on a Dionex UltiMate^®^ HPLC System (Thermo Fischer Scientific, Waltham, Massachusetts, United States). The eluted antibody was quantified via UV absorbance and integration of peak areas. Bio-Rad’s Gel Filtration Standard #151–1901 served as a gel filtration calibration standard.

Charge variants of TCB mAb 1 were analyzed via capillary isoelectric focusing (CIEF) according to the manufacturer’s protocol (Bio-Techne, Minneapolis, MN, United States).

### LC–MS peptide map procedure for Hyl analytic

Expressed and purified (ProtA) antibodies were denatured and reduced with 6 M of guanidine and 16 mM of DTT at pH 7 and 37°C for 1 h. The denatured reduced protein was then carboxymethylated using 73 mM of IAA-C12 (Fluka) and then buffer-exchanged on a NAP-5 column (GE Healthcare Life Sciences) into 50 mM TRIS and 2 mM CaCl_2_ at a pH of 7.5 and digested by trypsin (Promega) at 37°C for 1 h. The digested samples were then analyzed via LC–MS/MS. Liquid chromatography was performed on a Waters ACQUITY UPLC (Waters) using a reversed-phase ACQUITY CSH C18 column, 1.7 μm, 130 A, 2.1 × 150 mm (Waters). The aqueous mobile phase (mobile phase A) contained 0.1% (v/v) formic acid (FA) in HPLC-grade water. The organic mobile phase (mobile phase B) contained 0.1% (v/v) FA in acetonitrile. The gradient that was utilized in this experiment used a two-step linear gradient of 1%–30% of mobile phase B from 2 min to 33 min and to 60% until 42 min, followed by an increase to 90% between 42.5 and 44.5 min and a decrease back to 50% between 44.6 min and 50 min, followed by a re-equilibration at 1% eluent B from 50 min to 56 min. The column temperature was set to 65°C.

UPLC was coupled to an Orbitrap Fusion^TM^ Mass Spectrometer (Thermo Scientific). MS1 spectra were acquired using the Orbitrap mass analyzer with a resolution of 120,000, while MS/MS data were acquired in the Orbitrap analyzer with a resolution of 50,000. The MS/MS event on the Orbitrap was repeated for the TopN precursor ions with a dynamic exclusion window of 4.5 s enabled. The resulting MS data were processed using Byos^TM^ and Byonic^TM^ software (Protein Metrics Inc.). Manual data interpretation was performed using Byologic^TM^ software (Protein Metrics Inc.). The MS/MS Byos^TM^ search settings include a precursor mass tolerance of 5 ppm and a fragment mass tolerance of 20 ppm. Enzyme specificity was set to fully specific, allowing for one missed cleavage. The quantification of relative Hyl (“Kox,” lysine oxidized)-modified tryptic peptides, considering missed cleavage products (0 mc and 1 mc), compared to unmodified peptides (“K”) was calculated as indicated in Equation 5:

$$rel.\;area\;Hyl\;modified=\frac{\left[area\;Kox\;\left(0\;mc\right)+area\;Kox\;\left(1\;mc\right)\right]}{\left[area\;K\;\left(0\;mc\right)+area\;K\;\left(1\;mc\right)\right]+\left[area\;Kox\;\left(0\;mc\right)+area\;Kox\;\left(1\;mc\right)\right]}.$$ (5)

## Data Availability

The original contributions presented in the study are included in the article/Supplementary Material, further inquiries can be directed to the corresponding author.
