# Supplementary material for: Procollagen-lysine 2-oxoglutarate 5-dioxygenases are responsible for 5R-hydroxylysine modification of therapeutic T-cell bispecific monoclonal antibodies produced by Chinese hamster ovary cells
Source: Front Bioeng Biotechnol. 2024 Oct 28;12:1414408. doi: 10.3389/fbioe.2024.1414408 (PMC11551027; doi:10.3389/fbioe.2024.1414408)
Supplement: Supplementary file 1 [file DataSheet1.docx]

Procollagen-Lysine 2-Oxoglutarate 5-Dioxygenases are Responsible for 5R-Hydroxylysine Modification of Therapeutic T-Cell Bispecific Monoclonal Antibodies Produced by Chinese Hamster Ovary Cells

Niels Bauer^1,2*^, Marco Boettger^1*^, Stella Papadaki^1^, Tanja A. Leitner^1^, Stefan Klostermann^3^, Hubert Kettenberger^1^, Guy Georges^1^, Vincent Larraillet^1^, Dino Gluhacevic von Kruechten^4^, Lars Hillringhaus^4^, Annette Vogt^1^, Simon Auslaender^1^ & Oliver Popp^1,^ *^#^*

*^1^Large Molecule Research, Roche Pharma Research and Early Development (pRED), Roche Innovation Center Munich, Penzberg, Germany; ^2^Cell Culture and Bioprocess Operations Department, Genentech Inc., South San Francisco, CA, USA; ^3^Data and Analytics, Roche Pharma Research and Early Development (pRED), Roche Innovation Center Munich, Penzberg, Germany; ^4^Special Chemistry, Roche Diagnostics, Roche Innovation Center Munich, Penzberg, Germany*

**These authors contributed equally*

*^#^corresponding author, Dr. Oliver Popp,* [*oliver.popp@roche.com*](mailto:oliver.popp@roche.com)*, Phone: +49 8856 60 18420*

# Supplements

**Table S1 sgRNA sequences and verification primers used in this study.** sgRNA with highest knock-out score is highlighted in bold. (ch: Chinese hamster); na: not available

| **Target** | **sgRNA Name** | **sgRNA Sequence** | **Knock-out score** |
| --- | --- | --- | --- |
| chPLOD1 | **PLOD1_1** | **5’ - TAAGAGTTCCCGGGGCCCCG - 3’** | **98** |
|  | PLOD1_2 | 5’ - AACTCATCTACCCCGACCGG - 3’ | 96 |
|  | PLOD1_3 | 5’ - CGCTTGCCATCAGACACCGT - 3’ | 13 |
| chPLOD2 | **PLOD2_1** | **5’ - GTGGCCGGATAAGCGACTCG - 3’** | **97** |
|  | PLOD2_2 | 5’ - GTTTACCAATGTGCACTACA - 3’ | 86 |
|  | PLOD2_3 | 5’ - AAACGCTACCTGAATTCTGG - 3’ | na |
| chPLOD3 | PLOD3_1 | 5’ - GATGTTGCTCGAACAGTTGG - 3’ | na |
|  | **PLOD3_2** | **5’ - GGAGAAATATGCAAACCGGG - 3’** | **94** |
|  | PLOD3_3 | 5’ - CAAATTGCTGGTGATCACCG - 3’ | na |
| chJMJD4 | JMJD4_1 | 5’ - TGGGCCCCGCATAGACAAAG - 3’ | 83 |
|  | **JMJD4_2** | **5’ - CTGTAGGTCTCCCTTCCATG - 3’** | **95** |
|  | JMJD4_3 | 5’ - GCCTCCTGTATGACTTCAAG - 3’ | 69 |
| chJMJD6 | JMJD6_1 | 5’ - TGAATCCCGGTTCCAGAACG - 3’ | 91 |
|  | **JMJD6_2** | **5’ - TCCAGGCACTCGTTCCCAGA - 3’** | **96** |
|  | JMJD6_3 | 5’ - AAGGTGACCCGAGAAGAAGG - 3’ | 50 |
| chJMJD7 | **JMJD7_1** | **5’ - GCACAGCACGAGGTACACTG - 3’** | **97** |
|  | JMJD7_2 | 5’ - CAATCCCGGTAGAAGCAGAG - 3’ | 83 |
|  | JMJD7_3 | 5’ - AGCCATAGTGGGCTCCACGG - 3’ | 87 |


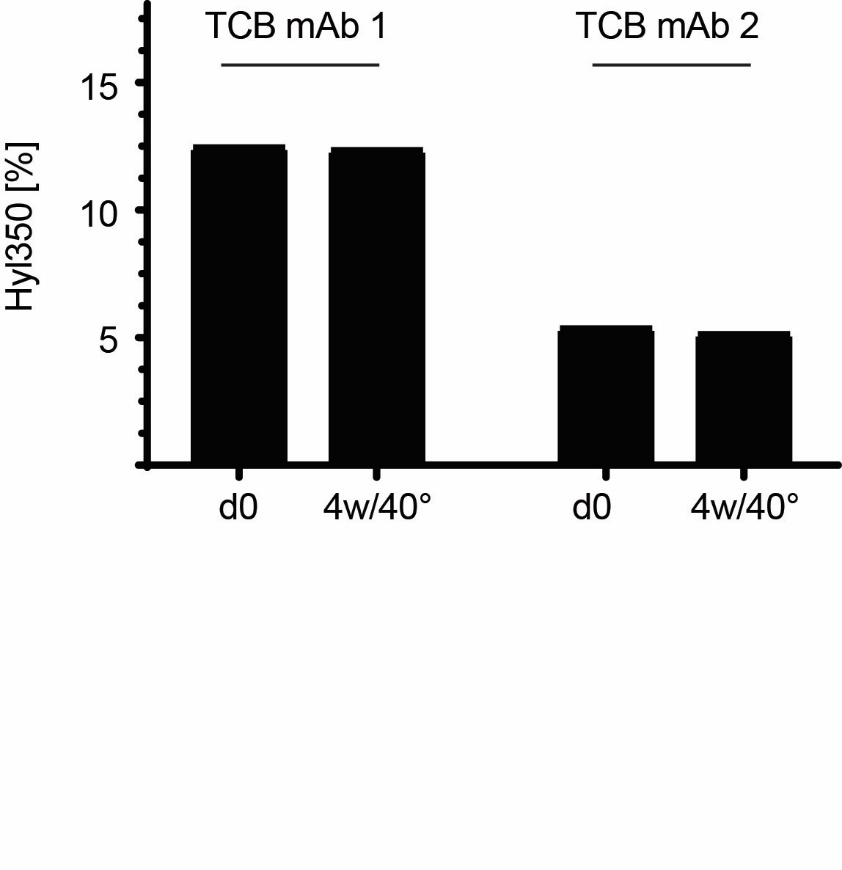


**Supplementary Figure S1 Hyl modification level is stable under stress conditions.** TCB mAb 1 and TCB mAb 2 Hyl levels at the start (d0) and after 4 weeks at 40°C in PBS buffer show no difference.


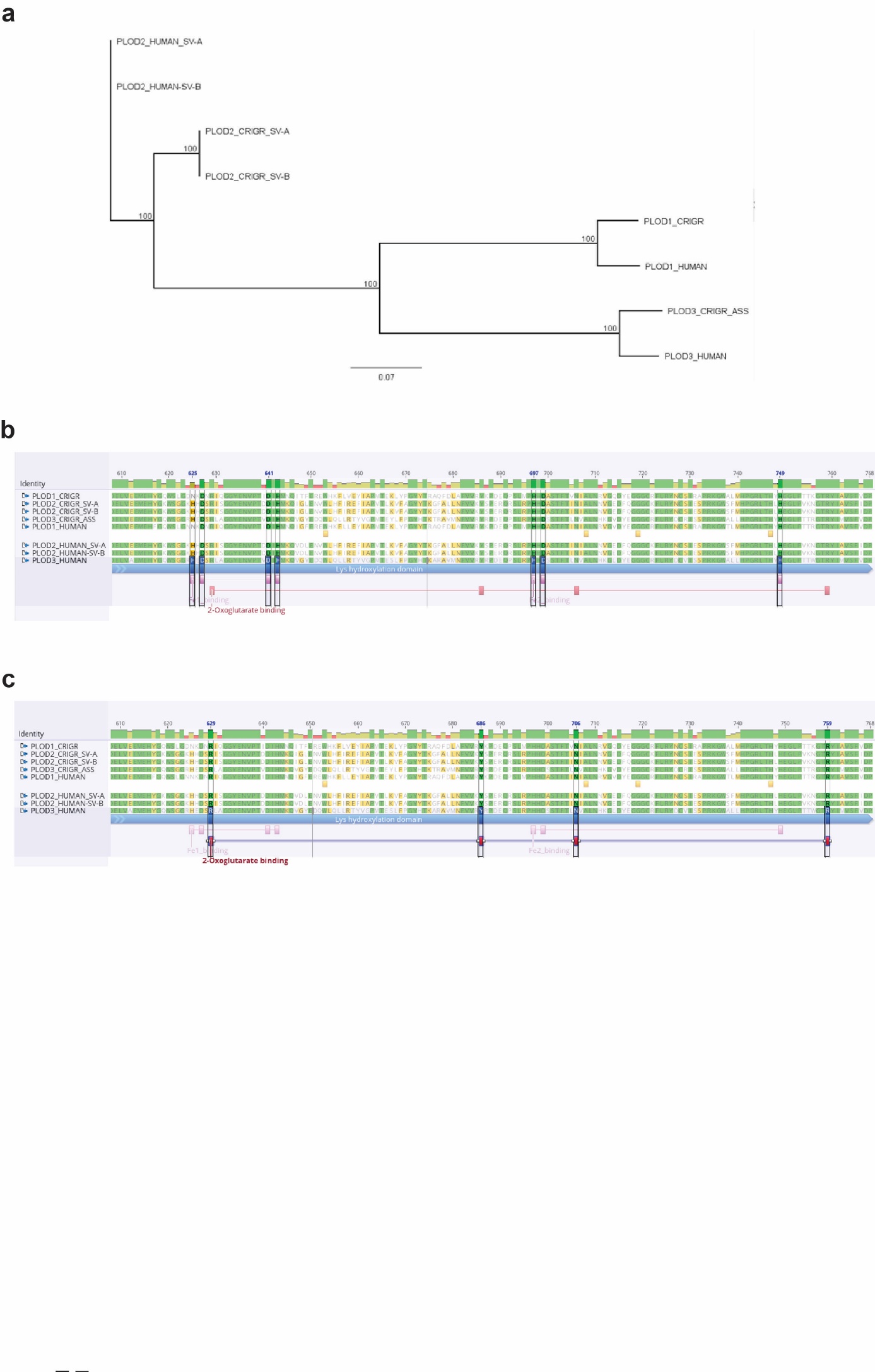


**Supplementary Figure S2 Chinese Hamster PLODs are highly conserved to human orthologues.** a) Hierarchical clustering analysis of Chinese hamster and human PLOD protein sequence suggest that PLODs of both species are highly conserved. b) Fe^2+^ and c) 2OG binding sites of Chinese hamster


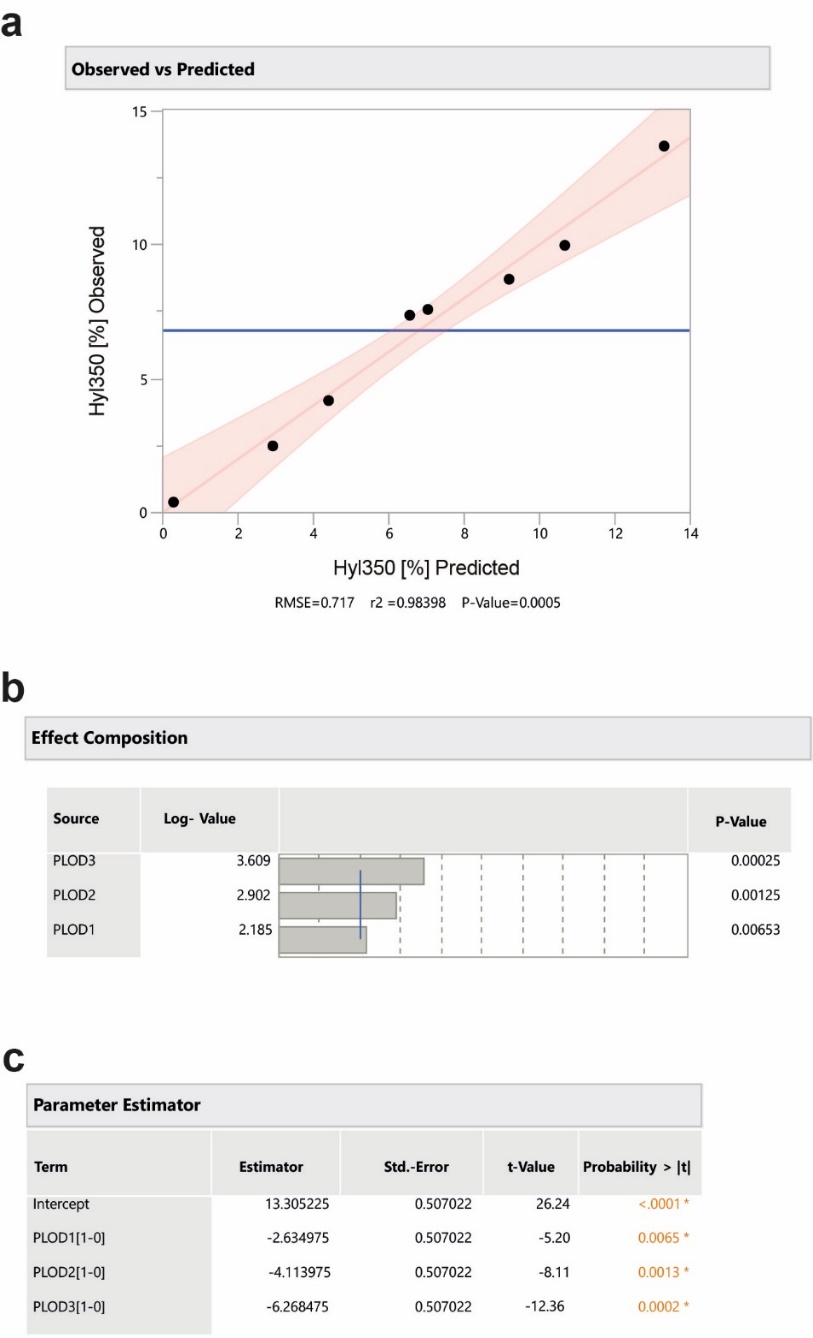


**Supplementary Figure S3 PLOD3 is main contributor to Hyl modification in TCB mAbs.** a) Multiregression model on observed “PLOD Contribution Study” Hyl modification values. The respective RMSE, r2 and P-Value indicating that the data can be described by a valid, statistically significant model. b) The effect composition analysis revealed the importance of PLOD3 over PLOD2 over PLOD1. c) The parameter estimator analysis show the respective estimator contribution and statistical probability. The analysis were conducted using JMP analysis tool.


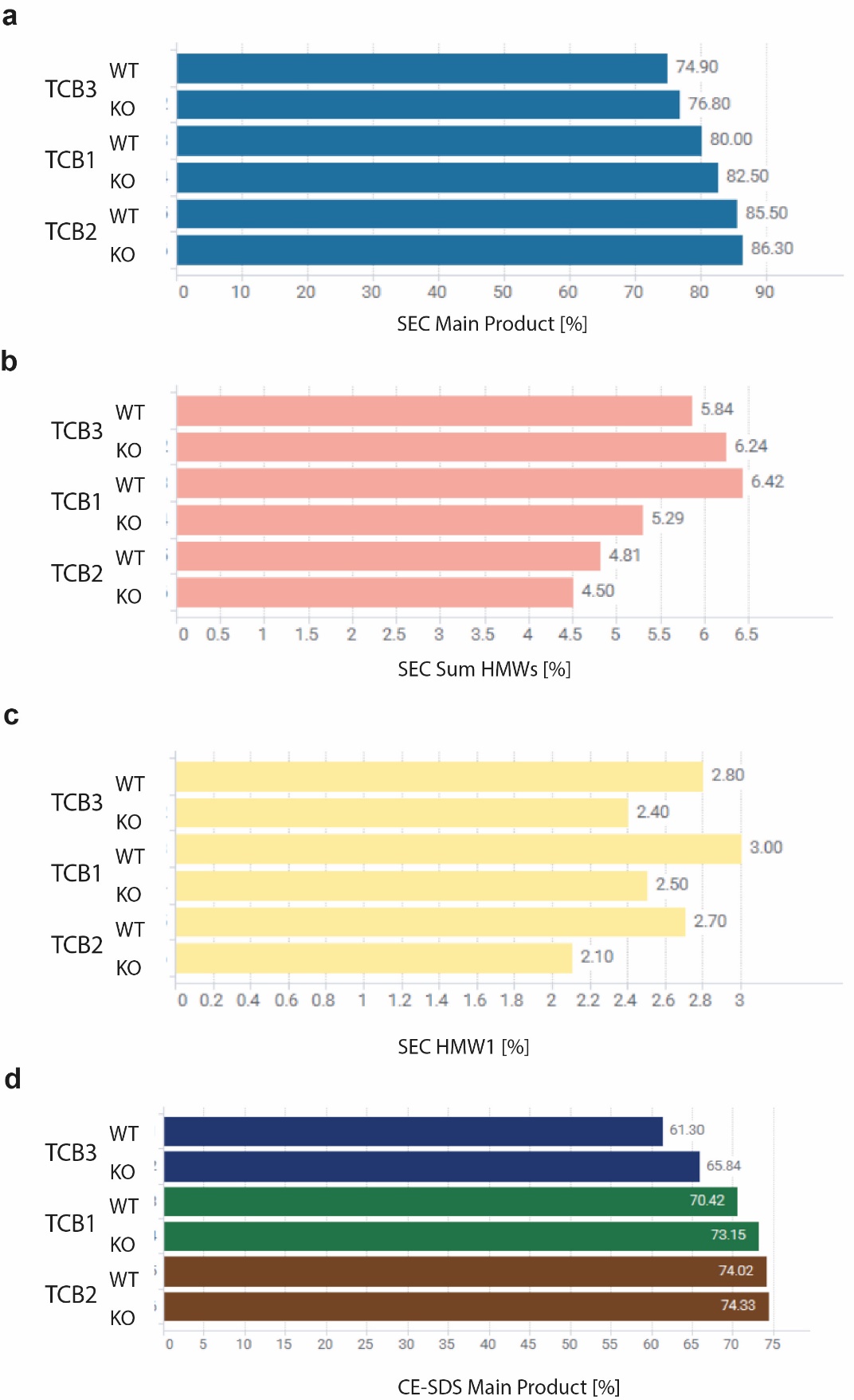


**Supplementary Figure S4 PLOD KO improves product quality of TCB mAb expression cultures.** Cell culture supernatants of wild-type (wt) and CRISPR/Cas9 KO cultures for PLOD1-3 were purified by ProtA and subsequently assessed by SEC and CE-SDS. a) Main product, b) sum HMWs, and c) HMW1 suggest an improved product quality by CHO cell PLOD depletion. Same ProtA purified materials were analyzed by non-reducing CE-SDS (d) which supported the observation of improved product quality by SEC.


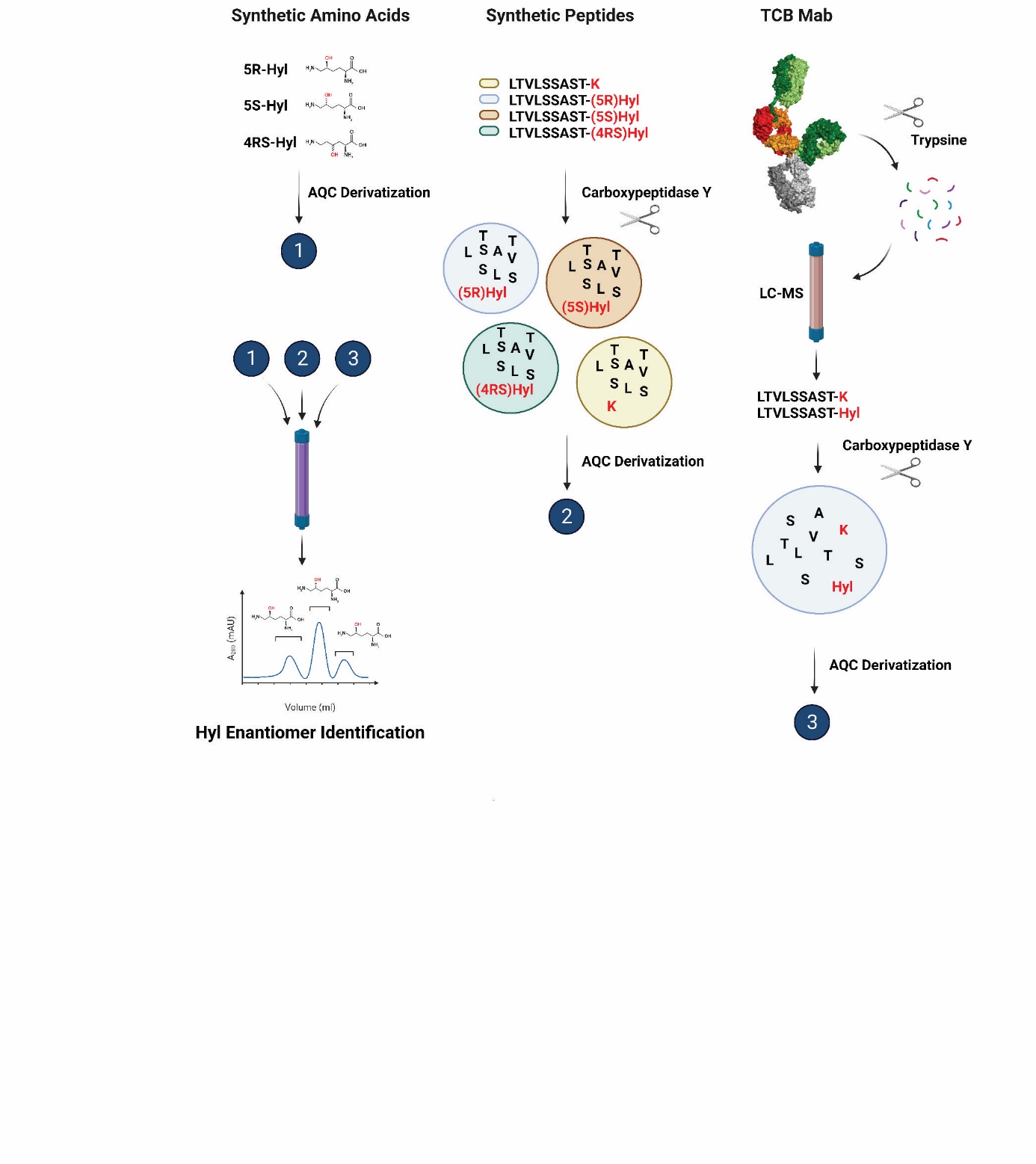


**Supplementary Figure S5 Analytical approach to identify TCB mAb modifying 2OG-dependent hydroxylase.**

**Supplementary Figure S6 PLOD1 sequence alignment human and Chinese hamster**

PLOD1_HUMAN  MRPLLLLALL GWLLLAEAKG DAKPEDNLLV LTVATKETEG FRRFKRSAQF FNYKIQALGL GEDWNVEKGT SAGGGQKVRL LKKALEKHAD KEDLVILFAD  100

PLOD1_CRIGR  MRPLLLLAPL AWLLLAQAKD DAKLEDNLLV LTVATKETEG FRRFKRSAQF FNYKIQALGL GEDWSVDSGP SAGGGQKVRL LKKALEKHAH KEDLVILFTD  100

PLOD1_HUMAN  SYDVLFASGP RELLKKFRQA RSQVVFSAEE LIYPDRRLET KYPVVSDGKR FLGSGGFIGY APNLSKLVAE WEGQDSDSDQ LFYTKIFLDP EKREQINITL  200

PLOD1_CRIGR  SYDVVFASGP RELLKKFQQA KSRVVFSAEE LIYPDRRLEA KYPTVSDGKR FLGSGGFIGY APNLNKLVAE WEGQDSDSDQ LFYTKIFLDP EKREQINISL  200

PLOD1_HUMAN  DHRCRIFQNL DGALDEVVLK FEMGHVRARN LAYDTLPVLI HGNGPTKLQL NYLGNYIPRF WTFETGCTVC DEGLRSLKGI GDEALPTVLV GVFIEQPTPF  300

PLOD1_CRIGR  DHRCRIFQNL DGALDEVVLK FEMGHVRARN LAYDTLPVVI HGNGPTKLQL NYLGNYIPRF WTFETGCTVC DEGLRSLKGI GDEALPTVLV GVFIEQPTPF  300

PLOD1_HUMAN  VSLFFQRLLR LHYPQKHMRL FIHNHEQHHK AQVEEFLAQH GSEYQSVKLV GPEVRMANAD ARNMGADLCR QDRSCTYYFS VDADVALTEP NSLRLLIQQN  400

PLOD1_CRIGR  LSLFFLRLLR LRYPQKRMRL FIHNHEQHHK LEVEKFLAEH GTEYQSVKLV GPEVRMANAD ARNMGADLCR QDQTCTYYFS VDADVALTEP DSLRLLIEQN  400

PLOD1_HUMAN  KNVIAPLMTR HGRLWSNFWG ALSADGYYAR SEDYVDIVQG RRVGVWNVPY ISNIYLIKGS ALRGELQSSD LFHHSKLDPD MAFCANIRQQ DVFMFLTNRH  500

PLOD1_CRIGR  KNVIAPLMTR HGRLWSNFWG ALSADGYYAR SEDYVDIVQG RRVGVWNVPY ISNIYLIKGS ALRAELQHVD LFHYSKLDAD MSFCANVRQQ EVFMFLTNRH  500

PLOD1_HUMAN  TLGHLLSLDS YRTTHLHNDL WEVFSNPEDW KEKYIHQNYT KALAGKLVET PCPDVYWFPI FTEVACDELV EEMEHFGQWS LGNNKDNRIQ GGYENVPTID  600

PLOD1_CRIGR  TFGHLLSLDN YQTTHLHNDL WEVFSNPEDW KEKYIHENYT KALEGKLVEM PCPDVYWFPI FTEAACDELV EEMEHYGQWS LGDNKDNRIQ GGYENVPTID  600

PLOD1_HUMAN  IHMNQIGFER EWHKFLLEYI APMTEKLYPG YYTRAQFDLA FVVRYKPDEQ PSLMPHHDAS TFTINIALNR VGVDYEGGGC RFLRYNCSIR APRKGWTLMH  700

PLOD1_CRIGR  IHMNQITFER EWHKFLVEYI APMTEKLYPG YYTRAQFDLA FVVRYKPDEQ PSLMPHHDAS TFTVNIALNR VGQDYEGGGC RFLRYNCSIR APRKGWALMH  700

PLOD1_HUMAN  PGRLTHYHEG LPTTRGTRYI AVSFVDP  727

PLOD1_CRIGR  PGRLTHYHEG LPTTKGTRYI AVSFVDP  727

**Supplementary Figure S7 PLOD2a sequence alignment human and Chinese hamster**

PLOD2_HUMAN_SV-A  MGGCTVKPQ. ....LLLLAL VLHPWNPCLG ADSEKPSSIP TDKLLVITVA TKESDGFHRF MQSAKYFNYT VKVLGQGEEW RGGDGINSIG GGQKVRLMKE   95

PLOD2_CRIGR_SV-A  MGGRRVRPGR LGLLLLLRGL ALLSWAPGLG AAEETPSRIP ADKLLVITVA TKENDGFHRF MNSAKYFNYT VKVLGQGQEW RGGDGINSIG GGQKVRLMKE  100

PLOD2_HUMAN_SV-A  VMEHYADQDD LVVMFTECFD VIFAGGPEEV LKKFQKANHK VVFAADGILW PDKRLADKYP VVHIGKRYLN SGGFIGYAPY VNRIVQQWNL QDNDDDQLFY  195

PLOD2_CRIGR_SV-A  AMAQYASQED LVILFTECFD VVFAGGPEEV LKKFQKTNHK IVFAADGILW PDKRLAEKYP VVHIGKRYLN SGGFIGYAPY ISHLVQEWNL QDNDDDQLFY  200

PLOD2_HUMAN_SV-A  TKVYIDPLKR EAINITLDHK CKIFQTLNGA VDEVVLKFEN GKARAKNTFY ETLPVAINGN GPTKILLNYF GNYVPNSWTQ DNGCTLCEFD TVDLSAVDVH  295

PLOD2_CRIGR_SV-A  TKVYIDPVKR EAFNITLDHK CKIFQALNGA TDEVVLKFEN GKSRVKNTFY ETLPVAINGN GPTKILLNYF GNYVPNSWTQ EHGCALCDFD TIDLSAVDVH  300

PLOD2_HUMAN_SV-A  PNVSIGVFIE QPTPFLPRFL DILLTLDYPK EALKLFIHNK EVYHEKDIKV FFDKAKHEIK TIKIVGPEEN LSQAEARNMG MDFCRQDEKC DYYFSVDADV  395

PLOD2_CRIGR_SV-A  PKVTIGVFIE QPTPFLPRFL NLLLSLDYPK EALKLFIHNK EVYHEKDIKV FFDKAKHEIS TIKIVGPEEN LSQAEARNMG MDFCRQDEKC DYYFSVDADV  400

PLOD2_HUMAN_SV-A  VLTNPRTLKI LIEQNRKIIA PLVTRHGKLW SNFWGALSPD GYYARSEDYV DIVQGNRVGV WNVPYMANVY LIKGKTLRSE MNERNYFVRD KLDPDMALCR  495

PLOD2_CRIGR_SV-A  VLTNPRTLKN LIEQNRKIIA PLVTRHGKLW SNFWGALSPD GYYARSEDYV DIVQGKRVGI WNVPYMANVY LIQGKTLRSE MSERNYFVRD KLDPDMALCR  500

PLOD2_HUMAN_SV-A  NAREMGVFMY ISNRHEFGRL LSTANYNTSH YNNDLWQIFE NPVDWKEKYI NRDYSKIFTE NIVEQPCPDV FWFPIFSEKA CDELVEEMEH YGKWSGGKHH  595

PLOD2_CRIGR_SV-A  NAREMGMFMY ISNRHEFGRL LSTANYNTSH LNNDLWQIFE NPVDWKEKYI NRDYSKIFTE SIVEQPCPDV FWFPIFSERA CDELVEEMEH YGKWSGGKHH  600

PLOD2_HUMAN_SV-A  DSRISGGYEN VPTDDIHMKQ VDLENVWLHF IREFIAPVTL KVFAGYYTKG FALLNFVVKY SPERQRSLRP HHDASTFTIN IALNNVGEDF QGGGCKFLRY  695

PLOD2_CRIGR_SV-A  DSRISGGYEN VPTDDIHMKQ IGLENVWLHF IREFIAPVTL KVFAGYYTKG FALLNFVVKY SPERQRSLRP HHDASTFTIN IALNNVGEDF QGGGCKFLRY  700

PLOD2_HUMAN_SV-A  NCSIESPRKG WSFMHPGRLT HLHEGLPVKN GTRYIAVSFI DP  737

PLOD2_CRIGR_SV-A  NCSIESPRKG WSFMHPGRLT HLHEGLPVKN GTRYIAVSFI DP  742

**Supplementary Figure S8 PLOD2b sequence alignment human and Chinese hamster**

PLOD2_HUMAN-SV-B  MGGCTVKPQ. ....LLLLAL VLHPWNPCLG ADSEKPSSIP TDKLLVITVA TKESDGFHRF MQSAKYFNYT VKVLGQGEEW RGGDGINSIG GGQKVRLMKE   95

PLOD2_CRIGR_SV-B  MGGRRVRPGR LGLLLLLRGL ALLSWAPGLG AAEETPSRIP ADKLLVITVA TKENDGFHRF MNSAKYFNYT VKVLGQGQEW RGGDGINSIG GGQKVRLMKE  100

PLOD2_HUMAN-SV-B  VMEHYADQDD LVVMFTECFD VIFAGGPEEV LKKFQKANHK VVFAADGILW PDKRLADKYP VVHIGKRYLN SGGFIGYAPY VNRIVQQWNL QDNDDDQLFY  195

PLOD2_CRIGR_SV-B  AMAQYASQED LVILFTECFD VVFAGGPEEV LKKFQKTNHK IVFAADGILW PDKRLAEKYP VVHIGKRYLN SGGFIGYAPY ISHLVQEWNL QDNDDDQLFY  200

PLOD2_HUMAN-SV-B  TKVYIDPLKR EAINITLDHK CKIFQTLNGA VDEVVLKFEN GKARAKNTFY ETLPVAINGN GPTKILLNYF GNYVPNSWTQ DNGCTLCEFD TVDLSAVDVH  295

PLOD2_CRIGR_SV-B  TKVYIDPVKR EAFNITLDHK CKIFQALNGA TDEVVLKFEN GKSRVKNTFY ETLPVAINGN GPTKILLNYF GNYVPNSWTQ EHGCALCDFD TIDLSAVDVH  300

PLOD2_HUMAN-SV-B  PNVSIGVFIE QPTPFLPRFL DILLTLDYPK EALKLFIHNK EVYHEKDIKV FFDKAKHEIK TIKIVGPEEN LSQAEARNMG MDFCRQDEKC DYYFSVDADV  395

PLOD2_CRIGR_SV-B  PKVTIGVFIE QPTPFLPRFL NLLLSLDYPK EALKLFIHNK EVYHEKDIKV FFDKAKHEIS TIKIVGPEEN LSQAEARNMG MDFCRQDEKC DYYFSVDADV  400

PLOD2_HUMAN-SV-B  VLTNPRTLKI LIEQNRKIIA PLVTRHGKLW SNFWGALSPD GYYARSEDYV DIVQGNRVGV WNVPYMANVY LIKGKTLRSE MNERNYFVRD KLDPDMALCR  495

PLOD2_CRIGR_SV-B  VLTNPRTLKN LIEQNRKIIA PLVTRHGKLW SNFWGALSPD GYYARSEDYV DIVQGKRVGI WNVPYMANVY LIQGKTLRSE MSERNYFVRD KLDPDMALCR  500

PLOD2_HUMAN-SV-B  NAREMTLQRE KDSPTPETFQ MLSPPKGVFM YISNRHEFGR LLSTANYNTS HYNNDLWQIF ENPVDWKEKY INRDYSKIFT ENIVEQPCPD VFWFPIFSEK  595

PLOD2_CRIGR_SV-B  NAREMTLQRE KDSPTPETIQ MLRPPKGMFM YISNRHEFGR LLSTANYNTS HLNNDLWQIF ENPVDWKEKY INRDYSKIFT ESIVEQPCPD VFWFPIFSER  600

PLOD2_HUMAN-SV-B  ACDELVEEME HYGKWSGGKH HDSRISGGYE NVPTDDIHMK QVDLENVWLH FIREFIAPVT LKVFAGYYTK GFALLNFVVK YSPERQRSLR PHHDASTFTI  695

PLOD2_CRIGR_SV-B  ACDELVEEME HYGKWSGGKH HDSRISGGYE NVPTDDIHMK QIGLENVWLH FIREFIAPVT LKVFAGYYTK GFALLNFVVK YSPERQRSLR PHHDASTFTI  700

PLOD2_HUMAN-SV-B  NIALNNVGED FQGGGCKFLR YNCSIESPRK GWSFMHPGRL THLHEGLPVK NGTRYIAVSF IDP  758

PLOD2_CRIGR_SV-B  NIALNNVGED FQGGGCKFLR YNCSIESPRK GWSFMHPGRL THLHEGLPVK NGTRYIAVSF IDP  763

**Supplementary Figure S9 PLOD3 sequence alignment human and Chinese hamster**

PLOD3_HUMAN      MTSSGPGPRF LLLLPLLLP. ...PAASASD RPRGRDPVNP EKLLVITVAT AETEGYLRFL RSAEFFNYTV RTLGLGEEWR GGDVARTVGG GQKVRWLKKE   96

PLOD3_CRIGR_ASS  MAASGPEPRL FLLLLLLLPP PLLPVASASD RSRGSSPVNP DKLLVITVAT AETEGYRRFL QSAEFFNYTV RTLGLGHEWR GGDVARTVGG GQKVRWLKKE  100

PLOD3_HUMAN      MEKYADREDM IIMFVDSYDV ILAGSPTELL KKFVQSGSRL LFSAESFCWP EWGLAEQYPE VGTGKRFLNS GGFIGFATTI HQIVRQWKYK DDDDDQLFYT  196

PLOD3_CRIGR_ASS  MEKYANREDM IIMFVDSYDV ILASSPAELL KKFVQSGSHL LFSAEGFCWP EWGLAEQYPE VGMGKRFLNS GGFIGFAPTI HQIVRQWKYK DDDDDQLFYT  200

PLOD3_HUMAN      RLYLDPGLRE KLSLNLDHKS RIFQNLNGAL DEVVLKFDRN RVRIRNVAYD TLPIVVHGNG PTKLQLNYLG NYVPNGWTPE GGCGFCNQDR RTLPGGQPPP  296

PLOD3_CRIGR_ASS  RLYLDPGLRE KLKLNLDHKS RIFQNLNGAL DEVVLKFDQN RVRIRNVAYD TLPVVVHGNG PTKLQLNYLG NYVPNGWTPQ GGCGFCNQNQ RTLPGGQPPP  300

PLOD3_HUMAN      RVFLAVFVEQ PTPFLPRFLQ RLLLLDYPPD RVTLFLHNNE VFHEPHIADS WPQLQDHFSA VKLVGPEEAL SPGEARDMAM DLCRQDPECE FYFSLDADAV  396

PLOD3_CRIGR_ASS  RVLLAVFVEQ PTPFLPRFLQ RLLFLDYPRD RVSLFLHNNE VYHEPHIADV WPQLQAHFSA AKLVGPEEAL SPGEARDMAM DSCRQDPKCE FYFSLDADAV  400

PLOD3_HUMAN      LTNLQTLRIL IEENRKVIAP MLSRHGKLWS NFWGALSPDE YYARSEDYVE LVQRKRVGVW NVPYISQAYV IRGDTLRMEL PQRDVFSGSD TDPDMAFCKS  496

PLOD3_CRIGR_ASS  LTNPETLRIL IEQNRKVIAP MLSRHGKLWS NFWGALSPDE YYARSEDYVE LVQRKRVGVW NVPYISQAYV IRGETLRTEL PQKEVFSGSD TDPDMAFCKS  500

PLOD3_HUMAN      FRDKGIFLHL SNQHEFGRLL ATSRYDTEHL HPDLWQIFDN PVDWKEQYIH ENYSRALEGE GIVEQPCPDV YWFPLLSEQM CDELVAEMEH YGQWSGGRHE  596

PLOD3_CRIGR_ASS  LRDKGIFLHL SNQHEFGRLL ATSRYDTDHL HPDLWQIFDN PVDWKEQYIH ENYSRALDGQ GLVEQPCPDV YWFPLLTEQM CDELVEEMEH YGQWSGGRHE  600

PLOD3_HUMAN      DSRLAGGYEN VPTVDIHMKQ VGYEDQWLQL LRTYVGPMTE SLFPGYHTKA RAVMNFVVRY RPDEQPSLRP HHDSSTFTLN VALNHKGLDY EGGGCRFLRY  696

PLOD3_CRIGR_ASS  DSRLAGGYEN VPTVDIHMKQ VGYEDQWLQL LRTYVGPMTE YLFPGYHTKT RAVMNFVVRY RPDEQPSLRP HHDSSTFTLN VALNHKGVDY EGGGCRFLRY  700

PLOD3_HUMAN      DCVISSPRKG WALLHPGRLT HYHEGLPTTW GTRYIMVSFV DP  738

PLOD3_CRIGR_ASS  DCRISSPRKG WALLHPGRLT HYHEGLPTTR GTRYIMVSFV DP  742

**
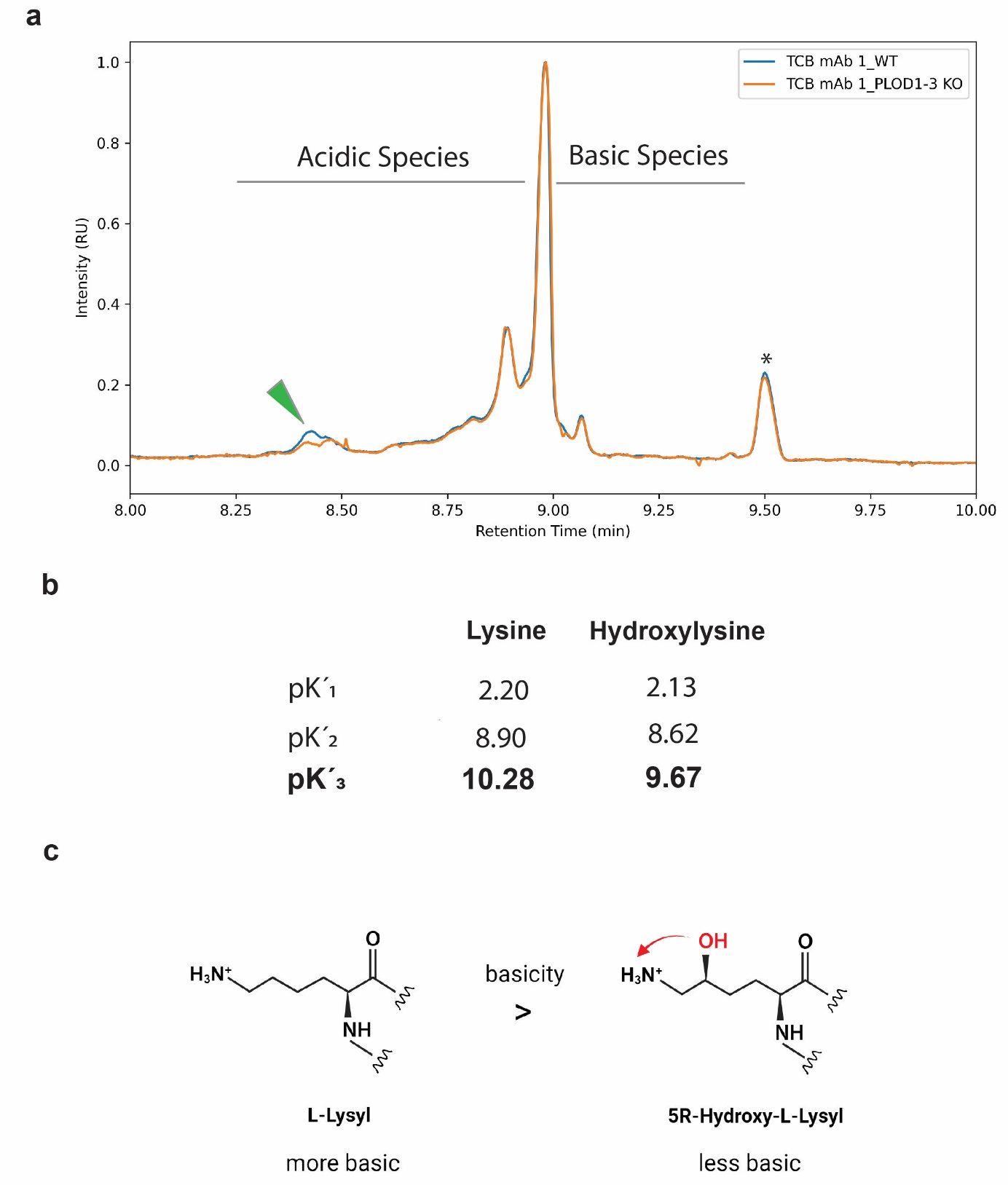
**

**Supplementary Figure S10 TCB mAb 1 expressed by PLOD1-3 KO CHO cells show a minor shift in reduced acidic species.** Cell culture supernatants of wild-type (wt) and CRISPR/Cas9 KO cultures for PLOD1-3 originating from TCB mAb 1 expressing CHO cells were purified by ProtA and subsequently assessed by cIEF (capillary isoelectric focusing) for charge variant analysis. a) Removal of Hyl modification in TCB mAb 1 by PLOD1-3 KO (<1% Hyl, orange chromatogram) showed a decreased acidic species pattern (green arrow head) compared to the WT control (> 12% Hyl, blue chromatogram). The chromatograms were normalized to the intensity of the main peak species. Asterisk marks internal standard used in cIEF. b) Dissociation constants of Lysine and Hydroxylysine according Klemperer et al.^69^ c) Schematic structure model of ionized L-Lysyl and 5R-Hydroxy-L-Lysyl in proteins with suggested basicity reduction of Hyl by interaction of the protonated epsilon amine and C5 hydroxyl group.

**End of Document**
